# Dissecting the interplay of model-based control, impulsivity and compulsivity on self-control in daily life

**DOI:** 10.1038/s41598-026-58046-4

**Published:** 2026-06-19

**Authors:** Kerstin Dück, Rebecca Overmeyer, Raoul Wüllhorst, Tanja Endrass

**Affiliations:** Faculty of Psychology Clinical Psychology and Addiction Research, Technische Universität Dresden, Chemnitzer Str. 46a 01187, 01062 Dresden, Germany

**Keywords:** Diseases, Neuroscience, Psychology, Psychology

## Abstract

Impaired self-control is linked to maladaptive behavior and psychopathology and may be shaped by transdiagnostic factors such as impulsivity and compulsivity. Both traits show associations with model-based control, which supports goal-directed behavior by representing long-term action consequences. To investigate how model-based control and personality traits relate to everyday self-control, we combined single-trial EEG data (modulation of the feedback-related negativity [FRN] and P3) from 236 participants during a two-step decision-making task with self-reported impulsivity (BIS-11) and compulsivity (OCI-R) and a seven-day ecological momentary assessment of daily-life self-control. Mixed-effects models revealed that desire enactment was more likely when desires were stronger and perceived conflicts weaker. The effect of model-based control varied with impulsivity and compulsivity: In individuals low in compulsivity, stronger modulations of the FRN, suggesting higher model-based control, were connected to fewer desire enactments, suggesting a protecting role of model-based control. This pattern reversed at high compulsivity levels, possibly due to greater conflict awareness but reduced behavioral regulation capacity. Impulsivity moderated compulsivity effects, such that model-based control consistently predicted reduced enactment at high impulsivity levels. These findings highlight how compulsivity and impulsivity shape the translation of cognitive control into everyday behavior and offer insights into mechanisms underlying self-control deficits.

**Supplementary Information:**

The online version contains supplementary material available at 10.1038/s41598-026-58046-4.

## Introduction

The average individual’s capacity for self-control is frequently challenged in daily life when deciding whether to give in to an immediate temptation or act in line with a longer-term personal goal. Self-control is needed when conflicting goals must be weighted^1^, such as sleeping in vs. getting up early to work. Beyond these everyday challenges, self-control is a key contributor to other domains of functioning, such as healthy diet^2^, academic success^3^, and mental health. Deficits in self-control have been linked to psychopathology such as obsessive-compulsive symptoms^4^ and disordered eating^5^. According to the Integrative Self-Control Theory^6^, self-control arises in situations of conflict between a desire and a higher-order goal. Whether or not a desire is enacted (self-control failure) depends on factors such as the subjective perception of the strength of the desire and the conflict and the motivation and capacity to exert control^7^. Despite its relevance for daily functioning and well-being, it remains unclear why individuals vary in their ability to exert self-control in everyday life.

Traditional models of self-control often focus on the inhibition of specific (desired) behaviors that contradict long-term goals^1^, but self-control also includes the initiation of goal-directed behavior that may be effortful or subjectively aversive^8^, e.g., studying or exercising. A key aspect of self-control appears to be an awareness of said long-term goals and how they could be achieved or threatened. Supporting this, Krönke et al.^9^ found that stronger value signaling in the ventromedial prefrontal cortex during reflection of long-term consequences was associated with fewer self-control failures. These results imply that self-control is connected to goal-directed behavior^10^, which relies on the mapping of actions with potential consequences and their fit with specific goals^11^. Such a mental model linking different actions, outcomes and specific states of the environment is the basis of model-based control^12–14^. Model-based control facilitates forward-planning and flexible, goal-directed behavior in complex environments at the cost of high computational demand^15–17^. In contrast, model-free control relies on past action-reward contingencies, which requires little cognitive effort but is linked to automatic and inflexible behavior^12,13,18^.

Model-based control is assessed in reinforcement learning paradigms such as two-step decision-making tasks^12,17^ where each trial consists of two decision-making stages. First-stage choices are mapped with different outcomes, i.e., second stages, in a probabilistic fashion, with each stimulus leading to one second stage with a higher probability (common transition) and to another with a lower probability (rare transition), respectively. In the second stage, participants choose between another set of stimuli, resulting in a reward or loss, with reward probabilities changing over time. Using this paradigm, model-based and model-free control can be differentiated by what informs first-stage choices: model-free control favors previously rewarded actions, regardless of the probability of subsequent rewards, i.e., transition type. Meanwhile, model-based control is guided by action-outcome mappings integrating outcome feedback with the transition structure, such that a reward following a rare transition would decrease the probability to repeat a stimulus choice, as the alternative stimulus is more likely to lead to the rewarding second stage in the next trial.

Neural markers of model-based control have been identified in electroencephalographic (EEG) studies of decision-making. The feedback-related negativity (FRN), a fronto-centrally negative component peaking around 200–350 ms after feedback presentation, has been associated with reward prediction errors (RPE), i.e., instances when outcomes do not occur as expected^19–21^, supporting behavioral adaptation^22^. The FRN has been connected to feedback valence, displaying higher amplitudes after negative vs. positive RPE^23–26^, while others relate the component to feedback magnitude^21,27^. Combining both features, higher FRN amplitudes for negative RPE have been found to vary further with feedback magnitude and probability, suggesting the FRN to reflect the integration of feedback information^28^. Its associations with reward and RPE link the FRN to model-free control^20^. However, as reward expectancies (i.e., RPE) depend on internal models of action-outcome contingencies, reflected in the learned transition structure, the FRN likely indexes model-based control as well^29^. Additionally, the centroparietal P3, beginning around 300 ms post-feedback, reflects stimulus relevance^30,31^ and model updating^32^. Given its association with feedback probability and RPE magnitude in decision-making paradigms, the component is thought to reflect the strength of task structure representations^29,33–35^. The P3 is thus associated with model-based control via the underlying mental model of the environment.

By strengthening the representation of potential outcomes, model-based control may facilitate self-control by amplifying desire-goal conflicts and enabling individuals to shift their focus away from an acute desire and towards a longer-term goal. Model-based control and self-control are further connected to impulsivity and compulsivity, two transdiagnostic traits relevant to cognitive control and psychopathology. Impulsivity is characterized as a predisposition towards rash, unplanned action without sufficient regard for possible negative consequences^36^. Conceptually, impulsivity favors responses in accordance with current desires instead of considering long-term goals, with a potential mechanism being reduced momentary awareness of conflicts in the moment such desires arise. Impulsivity has been negatively associated with self-control^37^ and used as a proxy for self-control deficits^38^. Further, impulsivity has been linked to a dysfunction of model-based control^29,39,40^. Compulsivity, defined as a tendency to repeat certain actions despite adverse consequences^41^ suggests the involvement of impaired self-control. In prior work, we found an altered P3 modulation in compulsive individuals during decision-making^29^, consistent with previous findings linking compulsivity to disrupted model-based control^35,42^.

Both traits, impulsivity and compulsivity, are relevant to a range of mental disorders, including obsessive-compulsive disorder (OCD)^43^, eating disorders^44,45^ and substance use disorder (SUD) and behavioral addictions^46,47^, all of which have been linked to dysfunctional model-based control^48^. Altered self-control and model-based control might contribute to the apparent risk for psychopathology with heightened impulsivity and compulsivity. Impulsivity and compulsivity show overlap in their neurobiological substrates^49^ and associations with clinical symptoms^50^, and interaction effects in cognitive domains such as performance monitoring^51^. However, they are often studied separately, leaving their combined influence on self-control unexplored.

In the present study, we investigated how model-based control, impulsivity, and compulsivity contribute to self-control in daily life in a sample of 236 individuals. We operationalized self-control with ecological momentary assessment (EMA) based on Hofmann et al.^7^ and as implemented by Wolff et al.^52^. Over the course of a week, participants received eight EMA prompts per day, asking them whether they experienced a desire (if so, its strength), whether it conflicted with personal goals (and if so, conflict strength), whether they tried to resist the desire, and whether they enacted the desire. To quantify model-based control, participants completed a two-step decision-making task during EEG recording. Single-trial regression analyses were used to compute the effect of the transition (rare vs. common) x RPE interaction – as a sign of a mental action-outcome model - on the EEG activity^29^. We assessed model-based control by examining how the effect of RPE on the FRN and P3 signals was modulated by the transition type of the trial, thereby capturing how feedback information is integrated by these components. Stronger model-based control is reflected in a greater modulation of FRN and P3 responses by transition type and RPE. These trial-wise neural indices were utilized as predictors in a brain-as-predictor approach^53^ together with impulsivity and compulsivity scores. We investigated their individual and interactive effects on self-control, focusing on all desires to account for possible momentary failures to detect desire–goal conflicts (self-monitoring failures^54^. Parallel analyses using EEG markers of inhibitory control (N2 and P3a) from a Go/NoGo task are detailed in the supplement.

Based on prior research and theoretical considerations, we formulated the following hypotheses. First, we expected situational characteristics to predict desire enactment: Specifically, stronger desires and weaker perceived conflict strength would increase the likelihood of enactment, replicating findings from Hofmann et al.^7^. Second, we hypothesized that EEG markers of model-based control, reflected in stronger single-trial effects of the interaction between RPE and transition type on the FRN and feedback-related P3 components, would be linked to reduced desire enactments, indicating more effective self-control. Third, we hypothesized that impulsivity and compulsivity, as transdiagnostic trait dimensions linked to both self-control and model-based control, would be associated with more frequent desire enactments. Given the established associations between these traits and both self-control and model-based control, we explored whether impulsivity and compulsivity would moderate the relationship between neural indices of model-based control and self-control in everyday life.

## Results

### Ecological momentary assessment

Of the 13,190 EMA questionnaires included in our analyses, participants reported experiencing a desire in 71.76% (*M* = 34.9, *SD* = 11.78). Of these instances, 39.86% (*M* = 13.91, *SD* = 8.53, 39.86%) were appraised as conflicting with a personal goal. Across all conflict-laden desires, participants reported enacting the desire, i.e., a self-control failure, in 56.26% of cases (*M* = 7.84, *SD* = 7.84; see Supplementary Table S4.1 for further detail on EMA results).

### EEG data: task effects

**Fig. 1:**
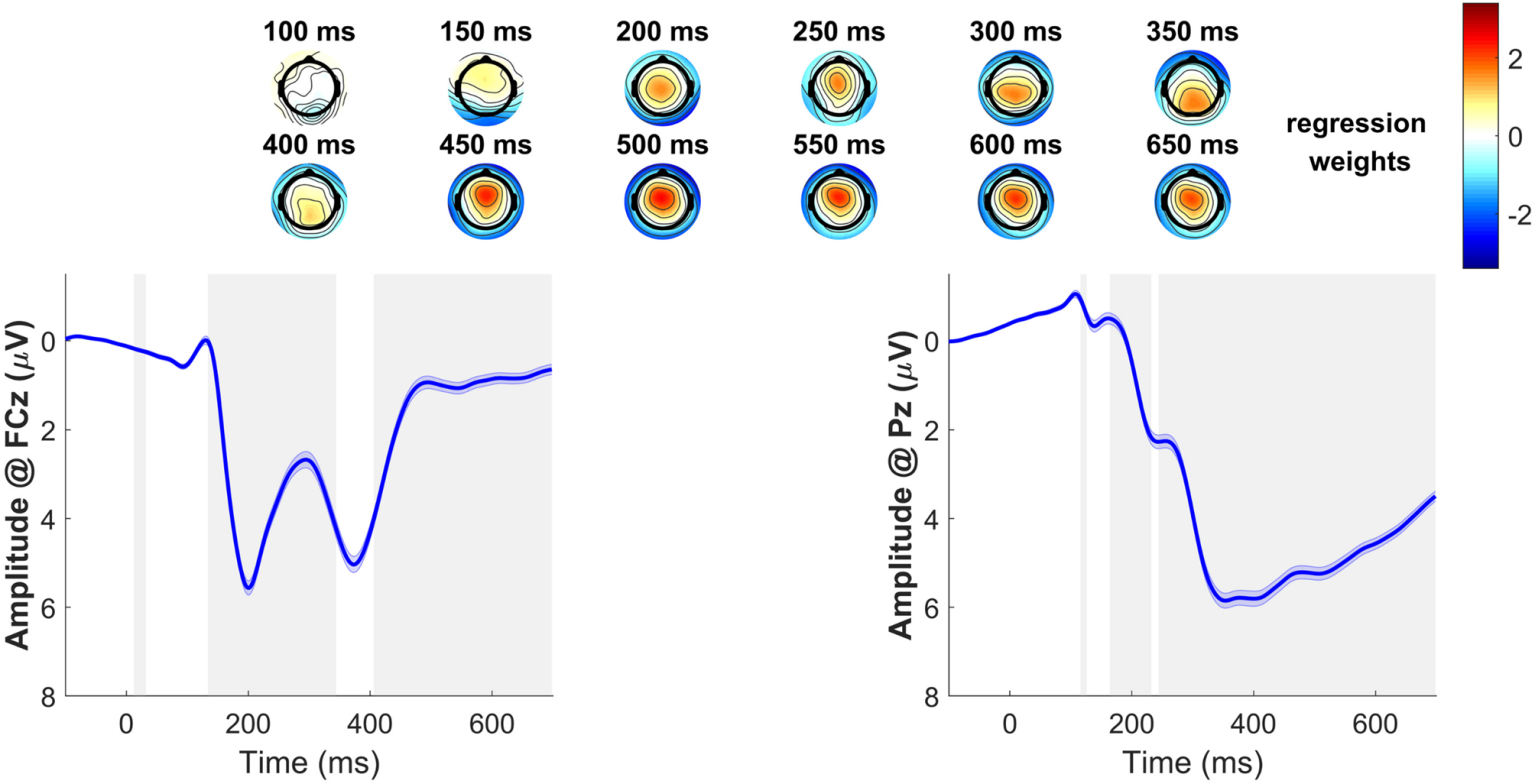
First-level effects of single-trial regression for the two-step task. First–second row: Topography of the *b* values for the first-level effect (100–650 ms) of the transition x RPE interaction. Third row: EEG time course at FCz (left) and Pz (right). Shading indicates SEM. EEG activity is locked to second-stage feedback presentation. Gray shading behind EEG activity indicates significance of regression weights (transition x RPE interaction; *p* < .05/2) after FDR-correction (at 5% FDR-level).

We analyzed the effects of trial characteristics on the EEG data using single-trial regression, yielding time-resolved regression weights for all electrodes (Fig. 1). We observed significant effects of the transition x RPE interaction, indicative of model-based control, on the EEG signals 300–350 ms and 350–500 ms after feedback onset. Specifically, robust regression effects of the transition x RPE interaction were evident in both the FRN (β_mean_ = 1.61, *p*_FDR-adjusted_ = < 0.001 at FCz) and P3 time-windows (β_mean_ = 1.74, *p*_FDR-adjusted_ = < 0.001 at Pz; averaged across participants). To quantify the strength of the model-based-control-related neural signals at the individual level, we extracted mean *b* values around the peak of each participant’s regression weight for the transition x RPE interaction within the respective time window for the FRN and P3 (FRN and P3 effects). All behavioral data as well as all task effects of the Go/Nogo task are detailed in supplements 3 and 4.

### Disentangling the effects on desire enactment

To examine how model-based-control-related EEG activity, impulsivity and compulsivity relate to self-control, we analyzed whether trial-by-trial markers of model-based control predict desire enactment, and how this relationship may be moderated by individual trait differences. EEG-based indices of model-based control were defined as each participant’s mean regression weights (*b-*values) for the transition x RPE interaction - reflecting a mental action-outcome model - in the FRN (FRN effect) and P3 (P3 effect) time windows. In logistic mixed-effects models, we regressed desire enactment onto conflict strength, desire strength, the FRN and P3 effects, and later added impulsivity and compulsivity scores. Desire strength and conflict strength were further modeled as random slopes to account for between-subject variability in their effects. In the base model (see supplement 5), stronger desires were more likely to be enacted (β = 5.16, 95% CI [4.87, 5.45], *p* < .001), whereas greater conflict strength reduced enactment probability (β = -1.52, 95% CI [-1.67, -1.37], *p* < .001). Neither the FRN nor the P3 effect significantly predicted desire enactment (see Supplementary Table S5.1). Adding trait impulsivity and compulsivity scores in the next model did not change the pattern: desire strength (β = 5.16, 95% CI [4.87, 5.45], *p* < .001) and conflict strength (β = -1.53, 95% CI [-1.68, -1.38], *p* < .001) remained robust predictors, whereas none of the EEG or trait variables reached significance (see Supplementary Table S5.2).

In the full model, impulsivity and compulsivity were included as interactions with the EEG effects (see Table 1). We observed significant moderating effects of compulsivity on the relationship between the FRN effect and desire enactment (β = 1.82, 95% CI [0.14, 3.49], *p* = .033) as well as a significant three-way interaction with impulsivity (β = -1.74, 95% CI [-3.38, -0.10], *p* = .037). Again, stronger desires (β = 5.16, 95% CI [4.87, 5.45], *p* < .001) and weaker conflict strength (β = -1.52, 95% CI [-1.67, -1.37], *p* < .001) were associated with higher enactment rates. The model explained substantial variance (conditional R^2^ = 0.77), with fixed effects alone (marginal R^2^) accounting for R^2^ = 0.65. Information criteria revealed a modest penalty of the full model compared to the base model (Δ_AIC_ = 4), indicating reduced parsimony (see Supplementary Table S5.3). However, because the interaction effects were theoretically central and substantively interpretable, we retained the full model.

**Table 1:** Full model predicting desire enactment.

Predictors	enactment		
	Odds Ratios	β [CI]	*p*
(Intercept)	0.04	-3.14 [-3.36, -2.92]	**< 0.001**
desire strength	174.25	5.16 [4.87, 5.45]	**< 0.001**
conflict strength	0.22	-1.52 [-1.67, -1.37]	**< 0.001**
FRN effect	0.59	− 0.52 [-1.71, 0.67]	0.392
P3 effect	2.53	0.93 [-0.48, 2.34]	0.195
FRN effect * impulsivity	1.53	0.43 [-0.70, 1.55]	0.457
FRN effect * compulsivity	6.15	1.82 [0.14, 3.49]	**0.033**
P3 effect * impulsivity	0.37	− 0.99 [-2.39, 0.42]	0.170
P3 effect * compulsivity	0.20	-1.59 [-3.28, 0.10]	0.066
FRN effect * impulsivity * compulsivity	0.18	-1.74 [-3.38, -0.10]	**0.037**
P3 effect * impulsivity * compulsivity	4.89	1.59 [-0.08, 3.26]	0.062

Random Effects			
σ^2^	3.29		
τ_00 participant_	0.30		
τ_11 participant.desire strength_	2.73		
τ_11 participant.conflict strength_	0.75		
ρ_01_	-0.66		
	0.39		
ICC	0.35		
N _participant_	236		
Observations	13,190		
Marginal R^2^ / Conditional R^2^	0.650 / 0.773		

*Notes.* CI = 95% confidence interval. FRN effect = mean *b* values for transition x RPE effect in the time-window for feedback-related negativity at FCz. P3 effect = mean *b* values for transition x RPE effect in the time-window for feedback-locked P3 at Pz. Impulsivity = sum score for Barratt Impulsiveness Scale 11. Compulsivity = sum score for Obsessive-Compulsive Inventory-Revised. *p* values < 0.05 are marked in boldface.

To compare these neural measures with a behavioral marker of MB control, we repeated our analyses using the weighting parameter *w*. Here, desire enactment was not predicted by *w* alone or its interaction with impulsivity or compulsivity scores (see Supplementary Table S5.4). We also examined whether the effects of EEG markers are specific to situations requiring self-control, fitting the same models using only data from conflict situations (see supplement 6). None of the inter-individual predictors (EEG or trait measures) significantly explained variance beyond desire strength and conflict strength. Lastly, we found only small-to-nonsignificant correlations between the inter-individual predictors in the regression model (see supplement 8).

Based on the significant interactions, we then explored the moderating roles of impulsivity and compulsivity on the relationship between the effect of the transition x RPE interaction on the FRN (FRN effect, as a neural marker of model-based control) and desire enactment. We used simple slope analysis^55^ to examine relationships across combinations of high, mean, and low levels (mean +- 1SD) of impulsivity and compulsivity scores to disentangle their moderating effects (see Table 2). Results indicate that the influence of model-based control on enactment was moderated by compulsivity: For individuals with low or average compulsivity scores, stronger FRN effects were associated with fewer desire enactments, suggesting more effective self-control. In contrast, among highly compulsive individuals, this relationship was reversed: Stronger FRN effects predicted more enactments, indicating less effective self-control, despite increased neural signals associated with model-based control.

**Table 2:** Simple slopes for the trend of the FRN effect on desire enactment depending on impulsivity and compulsivity scores.

		β (SE)	*p*
Low compulsivity			
impulsivity	low	-0.09 (0.14)	0.989
	mean	-0.08 (0.10)	
	high	-0.07 (0.11)	
Mean compulsivity			
impulsivity	low	0.12 (0.11)	0.067
	mean	-0.02 (0.08)	
	high	-0.17 (0.10)	
High compulsivity			
impulsivity	low	0.33 (0.17)	0.038
	mean	0.03 (0.11)	
	high	-0.26 (0.16)	
Low impulsivity			
compulsivity	low	-0.09 (0.14)	0.136
	mean	0.12 (0.11)	
	high	0.33 (0.17)	
Mean impulsivity			
compulsivity	low	-0.08 (0.10)	0.706
	mean	-0.02 (0.08)	
	high	0.03 (0.11)	
High impulsivity			
compulsivity	low	-0.07 (0.11)	0.541
	mean	-0.17 (0.10)	
	high	-0.26 (0.16)	

*Notes.* Trend effects for the FRN effect from a logistic mixed effects model predicting enactment. Estimates for the FRN effect are given for low, mean and high (mean +- 1SD) levels of impulsivity (sum scores for the Barratt Impulsiveness Scale 11) and compulsivity (sum scores for the Obsessive-Compulsive Inventory-Revised).

**Fig. 2:**
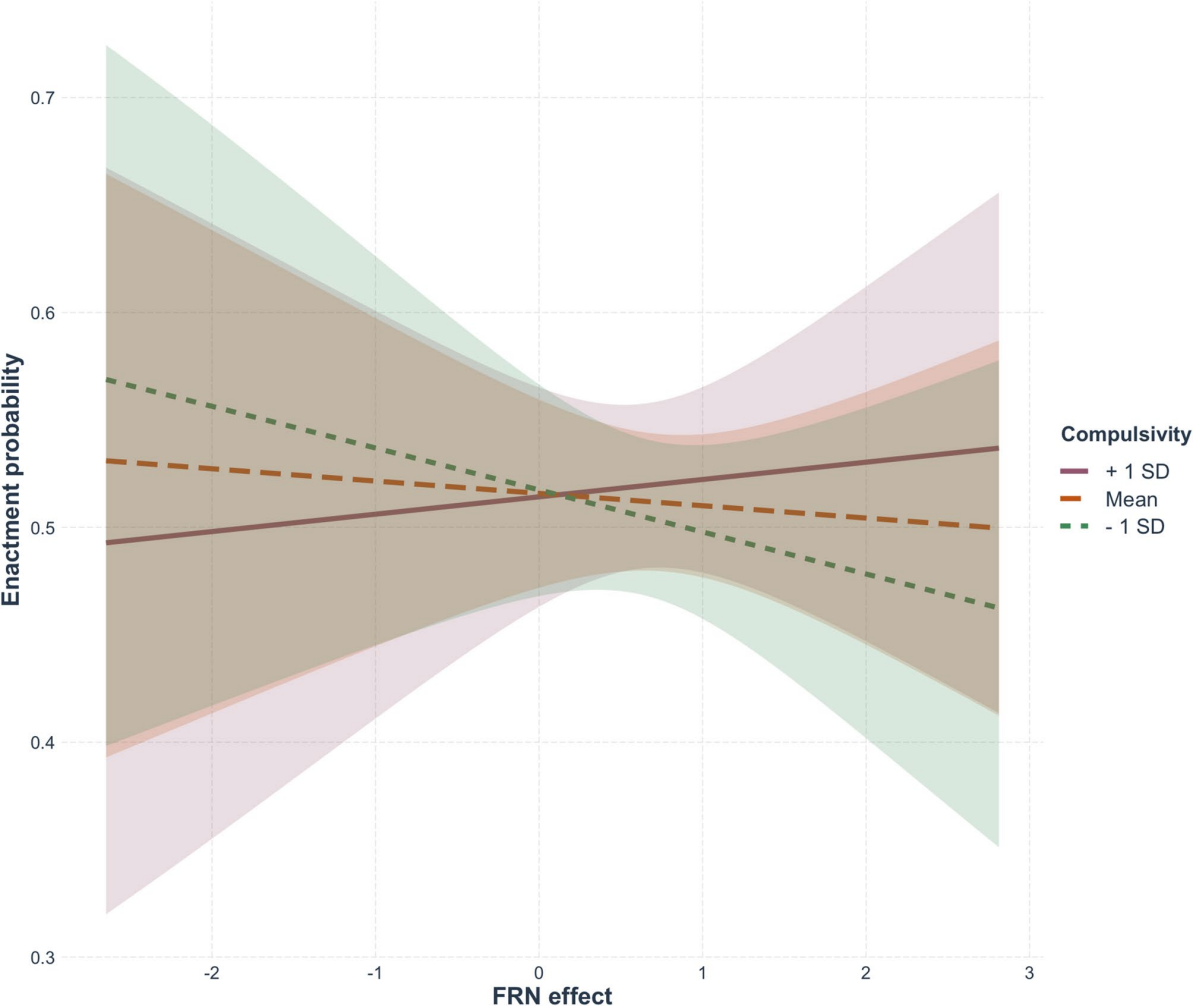
Compulsivity moderates the influence of the FRN effect on enactment probability. Regression of probability of desire enactment on the FRN effect (mean regression-based *b* values for transition x RPE effect in the time-window for feedback-related negativity at FCz), split for high (mean + 1 SD), moderate (mean) and low (mean – 1 SD) levels of compulsivity (OCI-R sum scores). Shading indicates 95% confidence intervals.

**Fig. 3:**
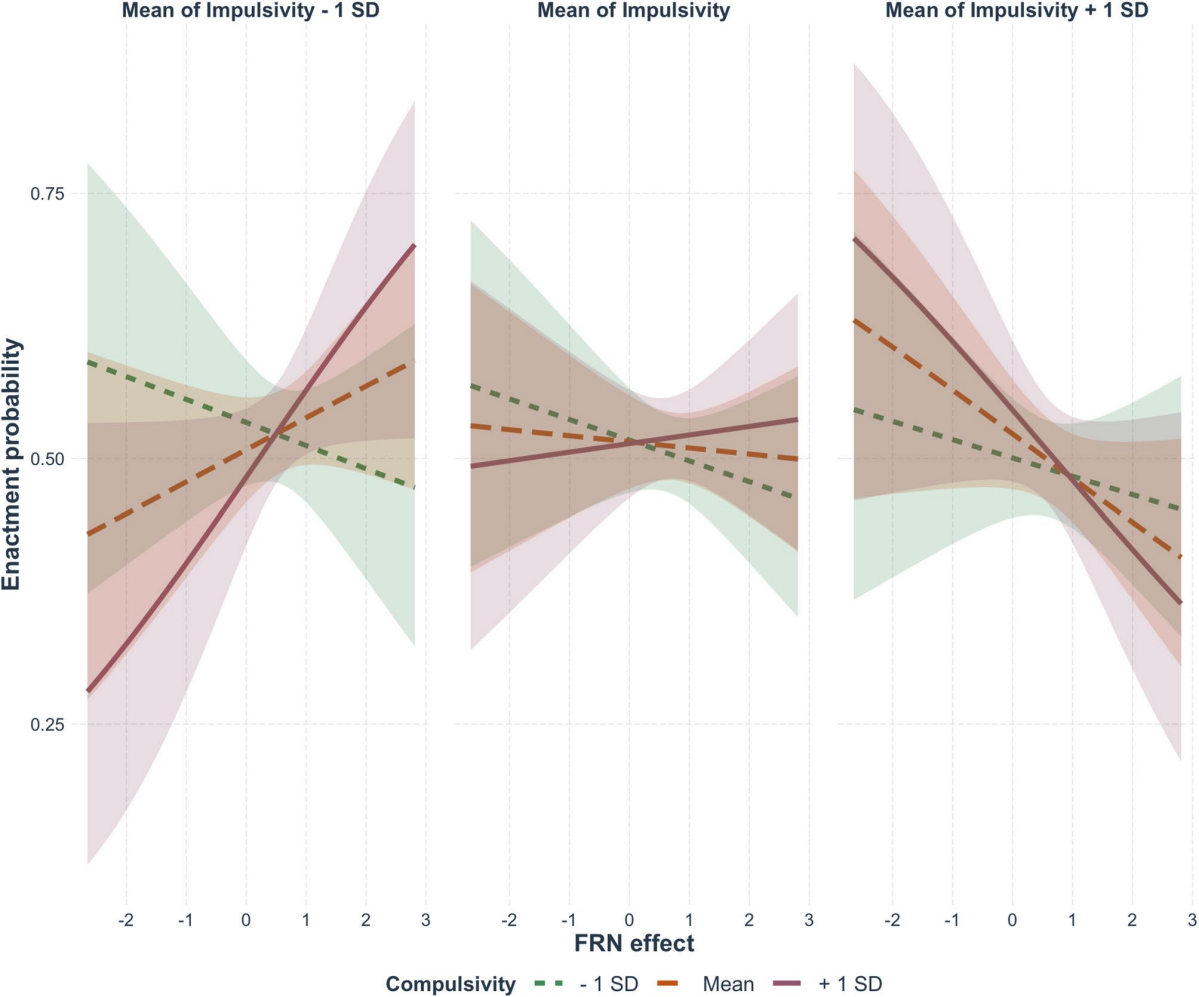
The moderating effect of compulsivity on the influence of the FRN effect is differentiated by impulsivity. Three-way interaction of the FRN effect, impulsivity and compulsivity on the probability of desire enactment. FRN effect reflects mean regression-based *b* values for transition x RPE effect in the time-window for feedback-related negativity at FCz for each participant. Simple slopes are split for low (mean – 1 SD), moderate (mean) and high (mean + 1 SD) levels of impulsivity (BIS-11 sum scores) and compulsivity (OCI-R sum scores). Shading indicates 95% confidence intervals. **A** Simple slopes of the FRN effect for varying compulsivity levels, moderated by impulsivity. **B** Simple slopes of the FRN effect for varying impulsivity levels, moderated by compulsivity.

Further exploration of the three-way interaction between the FRN effect, impulsivity, and compulsivity revealed that the aforementioned compulsivity-related moderation effect also depended on impulsivity. Specifically, when impulsivity was low or medium, compulsivity significantly altered the relationship between the FRN effect and enactment; As described above, at lower compulsivity, fewer enactments were associated with stronger FRN effects, whereas at higher compulsivity, more enactments were linked with stronger FRN effects. However, at high impulsivity levels, this moderating role of compulsivity disappeared, such that stronger FRN effects consistently predicted fewer enactment across all levels of compulsivity. In short, the interaction indicates that the negative association between a stronger FRN effect and enactment is moderated by compulsivity and impulsivity, reversing at higher compulsivity and low impulsivity (see Figs. 2 and 3).

We further explored whether individual differences in impulsivity and compulsivity, as well as their interaction with neural markers of model-based control, predicted the likelihood of reporting desires and conflicts. To this end, we estimated two logistic mixed-effects models in which the occurrence of a desire and the occurrence of a conflict were each regressed onto impulsivity and compulsivity scores, and their interactions with the FRN and P3 effects. The only significant predictors were compulsivity scores, which positively predicted both desire occurrence (β = 0.45, 95% CI [0.17, 0.74], *p* = .002) and conflict occurrence (β = 0.31, 95% CI [0.06, 0.57], *p* = .017). Thus, more compulsive individuals reported experiencing more frequent desires and more frequent conflicts between desires and personal goals (see supplement 7), as shown in recent findings from Overmeyer et al.^56^. No significant effects were observed for impulsivity or for any interaction terms involving the FRN and P3 effects.

## Discussion

In this study, we investigated how self-control in daily life is shaped by model-based control and the transdiagnostic personality traits impulsivity and compulsivity. Using a combination of EEG-derived markers of model-based control, ecological momentary assessment of desire enactment, and self-reported data, we aimed to bridge neural and behavioral levels of analysis using mixed-effects logistic regression models. As expected, situational factors were strong predictors of desire enactment. Desires were more likely to be enacted when they were perceived as stronger and less conflicting with personal goals. On their own, neither neural indices of model-based control nor trait-level variables predicted desire enactment. However, interaction effects indicated that the effects of model-based control as signaled by the modulation of the FRN was shaped by impulsivity and compulsivity, underscoring the dynamic interplay between cognitive control and personality in everyday behavior.

We used single-trial analysis of the psychophysiological data to establish signals of model-based control. The EEG signal during the two-step task was predicted by the interaction of trial type (common vs. rare) and RPE in the temporospatial windows of the FRN and the P3.

As suggested by our previous publication focusing on the main effects of trial type and RPE^29^, both the regression-based single-trial effects on the FRN and the P3 components likely reflect the use of internal action-outcome models to successfully combine reward information with the transition type indicating common vs. rare transitions. Beyond the FRN component itself as a reflection of reward^19–21^, the effect of the transition x RPE interaction on FRN-related activity indicates that the rewarding outcomes are weighted by the violation of expectations derived from internal models^29^. Of note, the FRN component itself may further be confounded by the reward positivity (RewP), an ERP thought to vary specifically with reward vs. neutral or negative outcomes^57,58^. Although the RewP is clearly observable as a difference wave, it has been suggested that it may be masked by the N2 component^59,60^, potentially accounting for the negative deflection seen in the FRN (see also our previous publication^28^ for the main effect of RPE on the FRN/RewP signal). However, as our analyses focus not on the basic ERP, but on how the EEG activity varies with the transition x RPE interaction, the FRN effect should not be substantially compromised. Similarly, the P3 varies with stimulus probability and their relevance for building and updating representations of the environment^25,32,61^ and is often considered to be linked directly to the internal task representation in decision-making paradigms^33,34^. The P3 effect thus likely captures a neural correlate of model-based control as well.

Transferring from lab-based neural indices to everyday behavior, our findings support Kotabe and Hofmann’s^6^ Integrative Theory of Self-Control, which conceptualizes self-control as arising from the coactivation of a desire and an at least partly incompatible higher order goal. In line with this framework and previous EMA studies^7,52^, desire strength promoted enactment based on expected rewards (e.g., the pleasant taste of a food item), while conflict strength reduced it by activating control. This replicates earlier findings and confirms that daily self-control fluctuates with the motivational structure of the situation.

Going beyond situational effects, we found that the relationship between model-based control and desire enactment varied systematically with compulsivity scores. For individuals with low compulsivity, stronger FRN effects (reflecting model-based control) were associated with fewer desire enactments. As expected, model-based control appears to facilitate self-control, possibly by strengthening the awareness of long-term goals and the potential consequences of actions. This interpretation resonates with previous findings linking reduced brain activity during anticipation of long-term effects of action alternatives with self-control failures in both a laboratory task and an EMA^9^.

However, among individuals high in compulsivity, stronger model-based control (as indexed by the FRN) was associated with more desire enactments. This seemingly paradoxical finding may reflect a misalignment between cognitive control and behavioral regulation in compulsivity. Compulsive individuals may process a heightened awareness of both their desires and conflicting goals, resulting in a strong desire for control but reduced sense of control over their environment^62,63^. Indeed, we found that higher compulsivity predicted more frequent reporting of desires and desire-goal conflicts, as previously established by Overmeyer at al.^56^. However, compulsive participants appear less capable of using said information to inform their decision-making, leading to more desire enactments. This apparent dysfunction is corroborated by various findings in OCD samples, such as a reduced adjustment in stimulus choices despite negative feedback^64,65^. Similarly, enhanced performance monitoring as signaled by the error-related negativity has been identified as an endophenotype of OCD^66^. However, despite an apparent neural hyperactivity, behavioral performance in the respective tasks is mostly equivalent to^67^ or even worse than in healthy controls^68^, suggesting that these enhanced neural signals do not amplify behavioral adaptation. Enhanced model-based control might increase the awareness of desires and conflicts, as goal-directed behavior requires knowledge of the respective goals as well as situations that might be beneficial or threatening towards their completion. Yet compulsive individuals show difficulties in using this mental model to guide their decision-making. The strong internal drive towards certain behaviors that characterizes compulsivity^41^ might surpass other information, thus further diminishing the influence of situational factors such as conflict strength^56^. Compulsivity is often linked to habits^69^, which are learned stimulus-response contingencies activating behavior irrespective of the current desirability of its outcome^70^. If compulsive individuals indeed have a reduced capacity to use information within their internal model, this might exacerbate their propensity to form habits, as a potential change in the value of an action due to a conflicting goal would be less likely to lead to an alternative behavior. This failure to translate goal-relevant information into action is characteristic of several compulsivity-related disorders, such as SUD or OCD^11,71,72^, where affected individuals often recognize the maladaptive nature of their behavior but still enact it.

The role of compulsivity further varied with impulsivity, as reflected in the three-way interaction. As stated above, stronger FRN effects were associated with fewer desire enactments at low levels of compulsivity, indicating a beneficial influence of model-based control on self-control. However, as compulsivity increased, this association reversed, suggesting that compulsive tendencies may amplify cognitive elaboration of desires and goal conflicts in ways that undermine the beneficial impact of model-based control. Alternatively, higher compulsivity may be associated with increased monitoring and reporting of self-control failures, which in turn could contribute to the reversed association between FRN effects of model-based control and enactment. Importantly, this effect was dependent on impulsivity levels. Among individuals with low to moderate impulsivity, compulsivity clearly differentiated the impact of model-based control on enactment. In contrast, in those with high impulsivity, stronger FRN effects consistently predicted fewer enactments, regardless of compulsivity. This may indicate that high impulsivity dampens compulsivity-related cognitive elaboration of self-control situations, allowing model-based control to exert a more direct inhibitory effect. Taken together, these findings suggest that the effectiveness of model-based control in everyday-self-control is not determined by its presence alone but critically shaped by the broader personality and motivational context in which it operates.

Interestingly, impulsivity alone did not predict desire enactment contrary to prior findings of reduced self-control with impulsivity^37^. This may be due to reduced awareness of both the desires themselves and resulting desire-goal conflicts. As impulsivity is connected to impaired model-based control^29,39,40^, a less robust mental model may contribute to this purported impaired use of contextual factors. Further, findings of an association between impulsivity and reduced awareness of motor intention^73^ and mindfulness^74–76^ support the idea that highly impulsive individuals may have difficulties recognizing desires as such before acting on them. Thus, potential impairments in behavioral regulation that we would expect in association with impulsivity may not correspond to our operationalization of daily-life self-control, resulting in a lack of direct association between self-control and impulsivity alone. Previous evidence linked impulsivity to (impaired) self-control using trait questionnaire data^37^. This supports recent arguments distinguishing trait and state self-control^77^ with trait impulsivity being more closely linked to stable tendencies such as conscientiousness^78^, while our EMA-based approach targets real-time enactment decisions.

One limitation of this study concerns the tradeoff between model complexity and parsimony. The full model produced slightly higher Akaike’s Information Criterion values than simpler nested models, indicating that the added complexity was not strongly supported by information-based model selection. Nevertheless, as the interaction terms were theoretically motivated and revealed substantial conditional relationships that were not apparent in simpler specifications, we retained the full model for inferential purposes. However, future work should examine the robustness of these effects across additional samples.

In conclusion, daily self-control is shaped by both situational features and individual differences. As expected, stronger desires and weaker goal conflicts increased the likelihood of desire enactment. Beyond these context effects, neural indicators of model-based control predicted self-control behavior in interaction with compulsivity and impulsivity. Our findings suggest that both impulsivity and compulsivity interfere with the effective deployment of model-based control on desire enactment, albeit through different mechanisms. Compulsivity appears to enhance the awareness of self-control-relevant situations while impeding behavioral adaptation, resulting in reduced self-control. This moderation emerged particularly in individuals low in impulsivity. High impulsivity, in the general population, may limit conflict awareness but allows model-based control to function more protectively when active. These findings highlight the complex interplay between cognitive control processes and personality in shaping self-control in daily life and may help explain why individuals with similar cognitive capacities differ in how they regulate behavior. Replication in clinical populations will be key to further understanding the translational relevance of these mechanisms for disorders marked by impaired self-regulation.

## Methods

### Participants

The current study is part of a larger project investigating different aspects of cognitive control (https://osf.io/ywnze/), involving data from 252 individuals from the general population. Inclusion criteria were age 18–45 years, native-level proficiency in German, and normal or corrected-to-normal vision. Participants were excluded if they reported a history of neurological disorder or severe head trauma; a lifetime diagnosis of bipolar disorder, borderline personality disorder, psychotic episodes, or severe alcohol use disorder; current eating disorder or severe episode of major depression; use of psychotropic medication within the last three months; or lifetime use of illicit substances more than twice a year or cannabis use more than twice a month. We excluded participants from the current analysis due to poor task compliance (two-step task: first-stage choices neither significantly predicted by model-based nor model-free control [*n* = 10] or task not completed [*n* = 2]; Go/Nogo task: multiple responses [*n* = 1]), or problems with EEG files [*n* = 3]. Thus, the final sample consisted of *N* = 236 participants (mean age = 25.15 years [*SD* = 5.10], 50% female, 95% with general higher education entrance qualification).

Participants gave written and informed consent and received financial compensation (80–100€) or course credit for participation in the study. The study was approved by the ethics committee at the University Hospital Carl Gustav Carus at the Technical University Dresden (EK 372092017) and conducted in accordance with the ethical guidelines of the Declaration of Helsinki.

### Procedure and measures

**Fig. 4:**
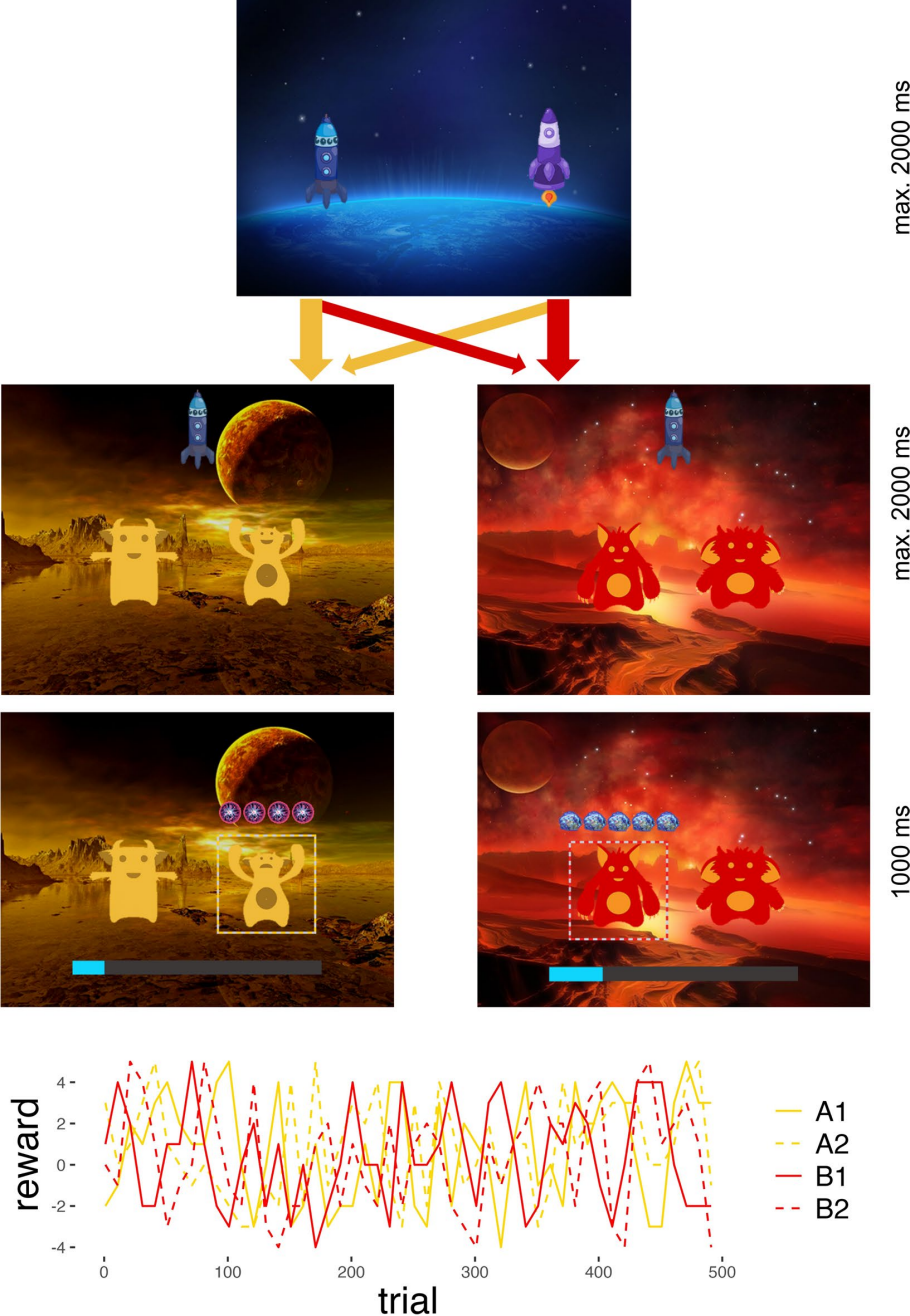
Two-step task measuring model-based control. Top: Schematic depiction of the task structure. On each trial, participants saw the first-stage display (2 spaceships) and one of two second stage displays (planets) with respective stimuli (aliens) and outcomes. First row: In stage one, participants chose one of two stimuli (spaceships), each probabilistically leading to one of two second stages (planets): with a probability of 80% (common transition; wide arrows; possible trial progression depicted on the left) or a probability of 20% (rare transition; narrow arrows; possible trial progression depicted on the right), respectively. Second row: Participants saw one of two second stages (planets) and choose between two stimuli (aliens) specific to the respective planet. Third row: The chosen stimulus was then marked. Each second-stage choice resulted in a gain or loss of points, shown above the stimulus, along with visual bar at the bottom indicating the participant’s cumulative point total. Bottom: Trajectories of rewards for each second-stage stimulus over time.

Participants completed the two-step task during an EEG session in the lab (see Fig. 4). Additional EEG tasks, as well as a neuropsychological test battery that was administered during a separate lab appointment, are not reported in the present paper. Lab sessions were scheduled at least seven days apart to accommodate a one-week ecological momentary assessment in between.

We additionally analyzed EEG data from a Go/Nogo task as a measure of inhibitory control, which yielded no relationship to self-control. Detailed descriptions of the Go/Nogo task, EEG processing, and corresponding results are provided in the supplement.

### Ecological momentary assessment

We employed ecological momentary assessment (EMA) to measure self-control in daily life. In each questionnaire, participants reported whether they had experienced a desire within the past hour, and if so, whether the desire conflicted with another goal, whether they tried to resist it, and whether they enacted the desire. When applicable, they categorized the type of desire and rated both desire strength and conflict strength on a 6-point Likert scale (see supplement 1 for details on the EMA questionnaire).

Participants received eight EMA prompts per day over the course of seven days (56 prompts total), signaled by an alarm signal on a study smartphone, which they were instructed to always keep with them. Prompts were delivered at randomized timepoints within a 14-hour window adapted to individual waking times (starting at 8, 9, or 10 a.m.), with a minimum of one hour between prompts. Each prompt could be postponed by up to 15 min. Thereafter, the entry was counted as missing. Participants received a financial bonus of 5€ if they completed at least 45 questionnaires. EMA was conducted using study smartphones equipped with a customized version of the movisensXS application (version 1.3.3; movisens GmbH, Karlsruhe, Germany). All non-study relevant functions on the devices were disabled for the duration of data collection.

We included all completed EMA questionnaires in our analyses to ensure statistical power. This encompassed both situations in which participants reported experiencing a desire and those in which they did not (in which case all variables were set to zero). On average, participants completed *M* = 48.63 (*SD* = 8.04) of the 56 EMA questionnaires, resulting in a total of 13,190 reported situations.

We further used the EMA data (*N* = 221) in another study within our research group, combining self-control with the error-related negativity (ERN) as an electrophysiological signal of performance monitoring. These analyses included the direct effects and interplay of impulsivity and compulsivity on desire enactment, as well as their moderating influence on desire strength, conflict strength and the ERN^56^.

### Two-step task

We assessed model-based control with a modified version of the two-step task^12,17^, which consists of two decision-making stages: In the first stage of each trial, participants were instructed to choose between two stimuli (cartoon drawings of spaceships). Each first-stage stimulus would lead them to one of two possible second stages (planets) with a fixed transition probability of 80% (common transition) or 20% (rare transition), respectively. Participants were then presented with the reached second stage (planet) and another pair of stimuli (aliens) specific to that stage. Second-stage stimulus choice resulted in gaining or losing of a variable number of points.

We constructed the reward probabilities for the second-stage stimuli with independent random walks with reflective bounds at + 5 and − 4 points. Participants were instructed that their chosen alien could yield up to five pieces of space treasure (adding points), up to four pieces of “anti-matter” (subtracting points), or nothing (no points). The goal was to maximize their total point score, which was transformed into a bonus of up to 5€ at the end of the task. Participants were aware of the probabilistic transition structure and the reward structure changing over time.

After a block of 25 practice trials, participants completed 500 trials divided into four blocks. First- and second-stage stimuli were shown until a response was made (stimulus choice, indicated with left or right index fingers; max. 2000 ms). Subsequently, the selected stimuli were marked for 500–800 ms, followed by either the second stage or a 1000 ms outcome display (icons indicating the respective number of space treasure or anti-matter) along with a visual bar indicating the participant’s total point count (see Fig. 4). Between trials, a black screen was shown for 300–800 ms.

In the two-step task, model-free and model-based learners can be differentiated by what informs their first-stage decision: model-free decisions are guided by previous rewards alone, favoring the repetition of rewarded actions regardless of the transition type. In contrast, model-based strategies integrate both reward and transition type of the previous trials. For example, after receiving a reward following a rare transition, model-based learners should switch to the alternative first-stage option, anticipating that it more likely leads to the rewarding second-stage state in the next trial. Accordingly, we investigated the interaction of transition and reward (indexed by RPE) as a sign of model-based control.

We employed computational modeling based on Kool et al.^17^, which yielded trial-based information such as the RPE as well as individual task parameters. See supplement 2 for a detailed description of model fitting and parameter estimation.

### Personality scales

Impulsivity was assessed with the Barratt Impulsiveness Scale (BIS-11)^79,80^, a 30-item self-report questionnaire that captures three facets of impulsivity: attentional, motor and non-planning impulsiveness. We used the BIS-11’s total sum score as an index of impulsivity. The BIS-11 has good internal consistency, with Cronbach’s alpha ranging from 0.77 to 0.88^80^.

Compulsivity was measured with the Obsessive-Compulsive Inventory-Revised (OCI-R)^81,82^. It consists of 18 self-report items assessing the severity of obsessive-compulsive symptoms across six domains: washing, checking, doubting, ordering, obsessing, hoarding, and neutralizing. We used the sum score as a measure of compulsivity. The OCI-R has shown good internal consistency (Cronbach’s alpha = 0.85;^86^.

### Data acquisition and analysis

Data processing was performed with MATLAB R2021a^83^ and the EEGlab toolbox, version 14.1.2b^84^ using the high-performance computing system (HPC) at the TU Dresden. Further regression analyses were performed with R (version 4.4.3)^85^.

### EEG recording and data reduction

EEG was recorded with Ag/AgCl electrodes from 61 sites of an equidistant electrode montage (EasyCap GmbH, Breitbrunn, Germany) and from three external position: two placed approximately 2 cm below each eye to monitor eye movements and one at the lower back to record the electrocardiogram. EEG was sampled at 500 Hz and amplified with two 32-channel BrainAmp amplifiers (Brain Products GmbH, Munich, Germany) and initially referenced to an electrode next to FCz. Continuous data was filtered offline between 0.1 and 30 Hz and submitted to an adaptive mixture independent component analysis (AMICA). We subsequently removed components containing eye movements and cardioballistic artifacts through visual inspection in combination with the ICLabel toolbox^86^. Data were subsequently re-referenced to average reference. Epochs ranging from − 200 to 800 ms around feedback onset were created and subjected to automated artifact rejection^87^: trials exceeding 5 SD from the mean probability distribution were excluded, with a minimum of one trial and a maximum of 5% of trials removed per participant. Baseline correction was applied in the 200 ms prior to feedback onset. Trials including reaction times below 100 ms in either stage of the two-step task were excluded.

### EEG single-trial analysis

To examine neural correlates of model-based control, we conducted single-trial regression analyses linking trial-wise EEG activity to task parameters of the two-step task. We regressed feedback-locked EEG activity from the second task stage at each electrode and time point onto transition type (common or rare), reward prediction error (RPE; signed), and their interaction (EEG ~ transition + RPE + transition x RPE) using robust regression. Resulting participant-level temporo-spatial maps of regression coefficients (*b* values) were then averaged over participants to investigate whether task variables explained significant variance in EEG activity. Our focus was on the interaction term (transition x RPE) as a neural marker of model-based control, which reflects the mental model through the effect of integration of feedback information on the EEG signal. We tested *b-*values against zero using two-tailed one-sample t-tests, correcting for multiple comparisons across electrodes and time points via false discovery rate (FDR)^88^, with an FDR-level of 5%. As we were specifically interested in event-related potentials related to feedback processing, we concentrated further analyses on electrode/time windows corresponding to the FRN and the P3.

Based on visual inspection and previous findings^29^, FRN-related regression-based single-trial effects were extracted from FCz in the 240–340 ms post-feedback interval^21^, and P3-related single-trial effects from Pz in the 350–500 ms window post-feedback^89^. Individual ERP-related *b*-values were averaged within +- 20 ms^25^ and +- 50 ms around the identified peak latency of the regression effect on the FRN and P3, respectively, to derive participant-specific indices (FRN and P3 effects) for further analysis.

Previous analyses of the same dataset focused on the main effects of transition and RPE on the EEG to assess the effects of behavioral model-based performance as well as impulsivity and compulsivity on feedback processing^29^.

### Regression model

We investigated how desire enactment was predicted by situational factors (desire strength and conflict strength) and individual characteristics (FRN and P3 effects as EEG indices of model-based control, and impulsivity and compulsivity) using logistic mixed-effects models implemented in the lme4 package in R (version 1.1–36)^90^. Variables were scaled before analysis. Model assumptions, such as data dispersion, were checked using base R features as well as the R package DHARMa (version 0.4.7)^91^. Model fit was assessed with the R package sjPlot (version 2.8.17)^92^.

First, we fit a base model to establish the effects of the situational variables and model-based control for all EMA questionnaires. In a logistic mixed-effects model, desire enactment was regressed onto desire strength, conflict strength and the FRN and P3 effects. Additionally, random intercepts and random slopes for desire strength and conflict strength were included to account for variance on the participant level. In a second model, impulsivity (Imp) and compulsivity (Comp) scores were added as predictors. Finally, in the full model, we allowed interactions between EEG signals of model-based control and both trait dimensions.

In R syntax, the full model was specified as follows:

Enactment ~ desire strength + conflict strength +.

FRN + FRN : Imp + FRN : Comp + FRN : Imp : Comp +.

P3 + P3 : Imp + P3 : Comp + P3 : Imp : Comp + (1 + desire strength + conflict strength| participant).

Significant interactions were further investigated with simple slopes analyses via the R package emmeans (version 1.10.7)^55^, focusing on how the relationships between EEG indices of model-based control (FRN and P3 effects) and desire enactment varied across combinations of high vs. low impulsivity and compulsivity scores.

We additionally fit the same mixed-effects models as described above using only data from EMA responses in which participants reported a conflict between the desire and a personal goal. In exploratory follow-up analyses, we also examined the likelihood of reporting a desire or a conflict as a function of trait impulsivity, compulsivity, and their interactions with EEG indices of model-based control (see also Overmeyer at al.,^56^. These were modelled as follows:

Desire (Conflict) ~ FRN : Imp : Comp + P3 : Imp : Comp + Imp + Comp + (1 + desire strength + conflict strength| participant).

Our preregistered analysis plan (https://osf.io/vjnhw/) originally aimed to delineate how the effects of desire strength and conflict strength were moderated by neural indicators of model-based (two-step task) and inhibitory control (Go/Nogo task) and impulsivity and compulsivity. We built models predicting desire enactment, which included the according three-way interactions (e.g., desire strength : FRN : Imp) and employed L1-penalized (LASSO) mixed-effects models to discern relevant predictors. However, the LASSO did not perform sufficiently in parameter selection and did not yield parsimonious models for further analyses. We thus revised the analysis strategy as detailed above to ensure feasibility and interpretability.

The data and R code for the regression analyses are available under https://osf.io/vjnhw/.

## Supplementary Information

Below is the link to the electronic supplementary material.

Supplementary Material 1

## Data Availability

Data and analysis routines are available under [https://osf.io/vjnhw/].

## References

[1] Inzlicht, M., Werner, K. M., Briskin, J. L. & Roberts, B. W. Integrating Models of Self-Regulation. *Ann. Rev. Psychol.* **72**, 319–345 (2021). 33017559 10.1146/annurev-psych-061020-105721

[2] Horwath, C. C., Hagmann, D. & Hartmann, C. The Power of Food: Self-control moderates the association of hedonic hunger with overeating, snacking frequency and palatable food intake. *Eat. Behav.* **38**, 101393 (2020). 32497904 10.1016/j.eatbeh.2020.101393

[3] Choi, I., Lim, S., Catapano, R. & Choi, J. Comparing two roads to success: Self-control predicts achievement and positive affect predicts relationships. *J. Res. Pers.* **76**, 50–63 (2018).

[4] Muhetaer, P., Leng, J. & Hu, P. Deficiency in Self-Control: Unraveling Psychological and Behavioral Risk Factors for Obsessive-Compulsive Symptoms in College Students. *Psychol. Res. Behav. Manag*. **17**, 1329–1338 (2024). 38524290 10.2147/PRBM.S456685 PMC10961077

[5] Eriksson, E., Ramklint, M., Wolf-Arehult, M. & Isaksson, M. The relationship between self-control and symptoms of anxiety and depression in patients with eating disorders: a cross-sectional study including exploratory longitudinal data. *J. Eat. Disord*. **11**, 21 (2023). 36788558 10.1186/s40337-023-00750-x PMC9930220

[6] Kotabe, H. P. & Hofmann, W. On Integrating the Components of Self-Control. *Perspect. Psychol. Sci.* **10**, 618–638 (2015). 26386000 10.1177/1745691615593382

[7] Hofmann, W., Baumeister, R. F., Förster, G. & Vohs, K. D. Everyday Temptations: An Experience Sampling Study of Desire, Conflict, and Self-Control. *J. Pers. Soc. Psychol.* **102**, 1318–1335 (2012). 22149456 10.1037/a0026545

[8] Gillebaart, M. The ‘Operational’ Definition of Self-Control. *Front. Psychol.* **9**, 1231 (2018). 30072939 10.3389/fpsyg.2018.01231 PMC6058080

[9] Krönke, K. M. et al. Predicting Real-Life Self-Control From Brain Activity Encoding the Value of Anticipated Future Outcomes. *Psychol. Sci.* **31**, 268–279 (2019). 10.1177/0956797619896357 32024421

[10] Boureau, Y. L., Sokol-Hessner, P. & Daw, N. D. Deciding How To Decide: Self-Control and Meta-Decision Making. *Trends Cogn. Sci.* **19**, 700–710 (2015). 26483151 10.1016/j.tics.2015.08.013

[11] Robbins, T. W., Gillan, C. M., Smith, D. G., de Wit, S. & Ersche, K. D. Neurocognitive endophenotypes of impulsivity and compulsivity: towards dimensional psychiatry. *Trends Cogn. Sci.* **16**, 81–91 (2012). 22155014 10.1016/j.tics.2011.11.009

[12] Daw, N. D., Gershman, S. J., Seymour, B., Dayan, P. & Dolan, R. J. Model-Based Influences on Humans’ Choices and Striatal Prediction Errors. *Neuron* **69**, 1204–1215 (2011). 21435563 10.1016/j.neuron.2011.02.027 PMC3077926

[13] Gläscher, J., Daw, N., Dayan, P. & O’Doherty, J. P. States versus Rewards: Dissociable Neural Prediction Error Signals Underlying Model-Based and Model-Free Reinforcement Learning. *Neuron* **66**, 585–595 (2010). 20510862 10.1016/j.neuron.2010.04.016 PMC2895323

[14] Daw, N. D., Niv, Y. & Dayan, P. Uncertainty-based competition between prefrontal and dorsolateral striatal systems for behavioral control. *Nat. Neurosci.* **8**, 1704–1711 (2005). 16286932 10.1038/nn1560

[15] Simon, D. A. & Daw, N. D. Neural Correlates of Forward Planning in a Spatial Decision Task in Humans. *J. Neurosci.* **31**, 5526–5539 (2011). 21471389 10.1523/JNEUROSCI.4647-10.2011 PMC3108440

[16] Doll, B. B., Simon, D. A. & Daw, N. D. The ubiquity of model-based reinforcement learning. *Curr. Opin. Neurobiol.* **22**, 1075–1081 (2012). 22959354 10.1016/j.conb.2012.08.003 PMC3513648

[17] Kool, W., Cushman, F. A. & Gershman, S. J. When Does Model-Based Control Pay Off? *Plos Comput. Biol.* **12**, e1005090 (2016). 27564094 10.1371/journal.pcbi.1005090 PMC5001643

[18] Dayan, P. & Niv, Y. Reinforcement learning: The Good, The Bad and The Ugly. *Curr. Opin. Neurobiol.* **18**, 185–196 (2008). 18708140 10.1016/j.conb.2008.08.003

[19] Miltner, W. H. R., Braun, C. H. & Coles, M. G. H. Event-Related Brain Potentials Following Incorrect Feedback in a Time-Estimation Task: Evidence for a Generic Neural System for Error Detection. *J. Cogn. Neurosci.* **9**, 788–798 (1997). 23964600 10.1162/jocn.1997.9.6.788

[20] Walsh, M. M. & Anderson, J. R. Learning from experience: Event-related potential correlates of reward processing, neural adaptation, and behavioral choice. *Neurosci. Biobehav Rev.* **36**, 1870–1884 (2012). 22683741 10.1016/j.neubiorev.2012.05.008 PMC3432149

[21] Sambrook, T. D., Goslin, J. A. & Neural Reward Prediction Error Revealed by a Meta-Analysis of ERPs Using Great Grand Averages. *Psychol. Bull.* **141**, 213–235 (2015). 25495239 10.1037/bul0000006

[22] Holroyd, C. B. & Coles, M. G. H. The neural basis of human error processing: Reinforcement learning, dopamine, and the error-related negativity. *Psychol. Rev.* **109**, 679–709 (2002). 12374324 10.1037/0033-295X.109.4.679

[23] Bai, Y., Katahira, K. & Ohira, H. Valence-separated representation of reward prediction error in feedback-related negativity and positivity. *Neuroreport* **26**, 157–162 (2015). 25634316 10.1097/WNR.0000000000000318

[24] von Borries, A. K. L., Verkes, R. J., Bulten, B. H., Cools, R. & de Bruijn, E. R. A. Feedback-related negativity codes outcome valence, but not outcome expectancy, during reversal learning. *Cogn. Affect. Behav. Neurosci.* **13**, 737–746 (2013). 24146314 10.3758/s13415-013-0150-1

[25] Kirsch, F., Kirschner, H., Fischer, A. G., Klein, T. A. & Ullsperger, M. Disentangling performance-monitoring signals encoded in feedback-related EEG dynamics. *NeuroImage* **257**, 119322 (2022). 35577025 10.1016/j.neuroimage.2022.119322

[26] Hoy, C. W., Steiner, S. C. & Knight, R. T. Single-trial modeling separates multiple overlapping prediction errors during reward processing in human EEG. *Commun. Biology*. **4**, 910 (2021). 10.1038/s42003-021-02426-1 PMC8302587 34302057

[27] Rawls, E. et al. Feedback-Related Negativity and Frontal Midline Theta Reflect Dissociable Processing of Reinforcement. *Front. Hum. Neurosci.* **13**, 452 (2020). 31998100 10.3389/fnhum.2019.00452 PMC6962175

[28] Bellebaum, C., Polezzi, D. & Daum, I. It is less than you expected: The feedback-related negativity reflects violations of reward magnitude expectations. *Neuropsychologia* **48**, 3343–3350 (2010). 20655319 10.1016/j.neuropsychologia.2010.07.023

[29] Dück, K., Wüllhorst, R., Overmeyer, R. & Endrass, T. On the effects of impulsivity and compulsivity on neural correlates of model-based performance. *Sci. Rep.* **14**, 21057 (2024). 39256477 10.1038/s41598-024-71692-w PMC11387645

[30] Donaldson, K. R., Oumeziane, B. A., Hélie, S. & Foti, D. The temporal dynamics of reversal learning: P3 amplitude predicts valence-specific behavioral adjustment. *Physiol. Behav.* **161**, 24–32 (2016). 27059320 10.1016/j.physbeh.2016.03.034 PMC5426362

[31] Friedman, D., Cycowicz, Y. M. & Gaeta, H. The novelty P3: an event-related brain potential (ERP) sign of the brain’s evaluation of novelty. *Neurosci. Biobehav Rev.* **25**, 355–373 (2001). 11445140 10.1016/s0149-7634(01)00019-7

[32] Donchin, E. & Coles, M. G. H. Is the P300 component a manifestation of context updating? *Behav. Brain Sci.* **11**, 357–374 (1988).

[33] Eppinger, B., Walter, M. & Li, S. C. Electrophysiological correlates reflect the integration of model-based and model-free decision information. *Cogn. Affect. Behav. Neurosci.* **17**, 406–421 (2017). 28050805 10.3758/s13415-016-0487-3

[34] Wurm, F., Ernst, B. & Steinhauser, M. The influence of internal models on feedback-related brain activity. *Cogn. Affect. Behav. Neurosci.* **20**, 1070–1089 (2020). 32812148 10.3758/s13415-020-00820-6 PMC7497542

[35] Seow, T. X. F. et al. Model-Based Planning Deficits in Compulsivity Are Linked to Faulty Neural Representations of Task Structure. *J. Neurosci.* **41**, 6539–6550 (2021). 34131033 10.1523/JNEUROSCI.0031-21.2021 PMC8318073

[36] Moeller, F. G., Barratt, E. S., Dougherty, D. M., Schmitz, J. M. & Swann, A. C. Psychiatric Aspects of Impulsivity. *Am. J. Psychiat*. **158**, 1783–1793 (2001). 11691682 10.1176/appi.ajp.158.11.1783

[37] Hamilton, K. R., Sinha, R. & Potenza, M. N. Self-reported impulsivity, but not behavioral approach or inhibition, mediates the relationship between stress and self-control. *Addict. Behav.* **39**, 1557–1564 (2014). 24508183 10.1016/j.addbeh.2014.01.003 PMC4222178

[38] Billen, E., Garofalo, C., Vermunt, J. K. & Bogaerts, S. Trajectories of Self-control in a Forensic Psychiatric Sample: Stability and Association with Psychopathology, Criminal History, and Recidivism. *Crim Justice Behav.* **46**, 1255–1275 (2019).

[39] Deserno, L. et al. Lateral prefrontal model-based signatures are reduced in healthy individuals with high trait impulsivity. *Transl Psychiatry*. **5**, e659–e659 (2015). 26460483 10.1038/tp.2015.139 PMC4930122

[40] Raio, C. M., Konova, A. B. & Otto, A. R. Trait impulsivity and acute stress interact to influence choice and decision speed during multi-stage decision-making. *Sci. Rep-uk*. **10**, 7754 (2020). 10.1038/s41598-020-64540-0 PMC7210896 32385327

[41] Luigjes, J. et al. Defining Compulsive Behavior. *Neuropsychol. Rev.* **29**, 4–13 (2019). 31016439 10.1007/s11065-019-09404-9 PMC6499743

[42] Voon, V. et al. Disorders of compulsivity: a common bias towards learning habits. *Mol. Psychiatr*. **20**, 345–352 (2015). 10.1038/mp.2014.44 PMC4351889 24840709

[43] Prochazkova, L. et al. Unpacking the role of self-reported compulsivity and impulsivity in obsessive-compulsive disorder. *CNS Spectr.* **23**, 51–58 (2018). 28487007 10.1017/S1092852917000244

[44] Carr, M. M., Wiedemann, A. A., Macdonald-Gagnon, G. & Potenza, M. N. Impulsivity and compulsivity in binge eating disorder: A systematic review of behavioral studies. *Prog Neuro-Psychopharmacol Biol. Psychiatry*. **110**, 110318 (2021). 10.1016/j.pnpbp.2021.110318 PMC8222068 33794320

[45] Lavender, J. M. et al. Facets of Impulsivity and Compulsivity in Women with Anorexia Nervosa. *Eur. Eat. Disord Rev.* **25**, 309–313 (2017). 28387426 10.1002/erv.2516 PMC7654514

[46] Lee, R. S. C., Hoppenbrouwers, S. & Franken, I. A. Systematic Meta-Review of Impulsivity and Compulsivity in Addictive Behaviors. *Neuropsychol. Rev.* **29**, 14–26 (2019). 30927147 10.1007/s11065-019-09402-x

[47] Ioannidis, K., Hook, R., Wickham, K., Grant, J. E. & Chamberlain, S. R. Impulsivity in Gambling Disorder and problem gambling: a meta-analysis. *Neuropsychopharmacology* **44**, 1354–1361 (2019). 30986818 10.1038/s41386-019-0393-9 PMC6588525

[48] Voon, V., Reiter, A., Sebold, M. & Groman, S. Model-Based Control in Dimensional Psychiatry. *Biol. Psychiatry*. **82**, 391–400 (2017). 28599832 10.1016/j.biopsych.2017.04.006

[49] Fineberg, N. A. et al. New developments in human neurocognition: clinical, genetic, and brain imaging correlates of impulsivity and compulsivity. *CNS Spectr.* **19**, 69–89 (2014). 24512640 10.1017/S1092852913000801 PMC4113335

[50] Fineberg, N. A. et al. Probing Compulsive and Impulsive Behaviors, from Animal Models to Endophenotypes: A Narrative Review. *Neuropsychopharmacol* **35**, 591–604 (2010). 10.1038/npp.2009.185 PMC3055606 19940844

[51] Overmeyer, R. & Endrass, T. Disentangling associations between impulsivity, compulsivity, and performance monitoring. *Psychophysiology***e14539**10.1111/psyp.14539 (2024). 10.1111/psyp.14539 38332720

[52] Wolff, M. et al. Action versus state orientation moderates the impact of executive functioning on real-life self-control. *J. Exp. Psychol. Gen.* **145**, 1635–1653 (2016). 27736135 10.1037/xge0000229

[53] Berkman, E. T. & Falk, E. B. Beyond Brain Mapping: Using Neural Measures to Predict Real-World Outcomes. *Curr. Dir. Psychol. Sci.* **22**, 45–50 (2013). 24478540 10.1177/0963721412469394 PMC3903296

[54] Hofmann, W. & Kotabe, H. A. General Model of Preventive and Interventive Self-Control. *Soc. Pers. Psychol. Compass*. **6**, 707–722 (2012).

[55] Lenth, R. V. *emmeans: Estimated Marginal Means, aka Least-Squares Means*. at (2024). https://rvlenth.github.io/emmeans/

[56] Overmeyer, R., Kräplin, A., Goschke, T. & Endrass, T. The association between the error-related negativity and self-control is moderated by impulsivity and compulsivity. *Commun. Psychol.* **4**, 62 (2026). 41896335 10.1038/s44271-026-00446-3 PMC13046855

[57] Holroyd, C. B., Pakzad-Vaezi, K. L. & Krigolson, O. E. The feedback correct‐related positivity: Sensitivity of the event‐related brain potential to unexpected positive feedback. *Psychophysiology* **45**, 688–697 (2008). 18513364 10.1111/j.1469-8986.2008.00668.x

[58] Proudfit, G. H. The reward positivity: From basic research on reward to a biomarker for depression. *Psychophysiology* **52**, 449–459 (2015). 25327938 10.1111/psyp.12370

[59] Baker, T. E. & Holroyd, C. B. Which Way Do I Go? Neural Activation in Response to Feedback and Spatial Processing in a Virtual T-Maze. *Cereb. Cortex*. **19**, 1708–1722 (2009). 19073622 10.1093/cercor/bhn223

[60] Holroyd, C. B. *in Errors, conflicts, and the brain: Current opinions on performance monitoring* 211–218 (Max Planck Institute, 2004).

[61] Kirschner, H., Fischer, A. G. & Ullsperger, M. Feedback-related EEG dynamics separately reflect decision parameters, biases, and future choices. *NeuroImage* **259**, 119437 (2022). 35788041 10.1016/j.neuroimage.2022.119437

[62] Moulding, R. & Kyrios, M. Desire for Control, Sense of Control and Obsessive-Compulsive Symptoms. *Cogn. Ther. Res.* **31**, 759–772 (2007).

[63] Froreich, F. V., Vartanian, L. R., Grisham, J. R. & Touyz, S. W. Dimensions of control and their relation to disordered eating behaviours and obsessive-compulsive symptoms. *J. Eat. Disord*. **4**, 14 (2016). 27144009 10.1186/s40337-016-0104-4 PMC4853853

[64] Endrass, T., Koehne, S., Riesel, A. & Kathmann, N. Neural Correlates of Feedback Processing in Obsessive–Compulsive Disorder. *J. Abnorm. Psychol.* **122**, 387–396 (2013). 23421527 10.1037/a0031496

[65] Marzuki, A. A. et al. Association of Environmental Uncertainty With Altered Decision-making and Learning Mechanisms in Youths With Obsessive-Compulsive Disorder. *JAMA Netw. Open.* **4**, e2136195 (2021). 34842925 10.1001/jamanetworkopen.2021.36195 PMC8630570

[66] Riesel, A. The erring brain: Error-related negativity as an endophenotype for OCD—A review and meta‐analysis. *Psychophysiology* **56**, e13348 (2019). 30838682 10.1111/psyp.13348

[67] Endrass, T. & Ullsperger, M. Specificity of performance monitoring changes in obsessive-compulsive disorder. *Neurosci. Biobehav Rev.* **46**, 124–138 (2014). 24747486 10.1016/j.neubiorev.2014.03.024

[68] Hanna, G. L. et al. Altered Error Monitoring and Decreased Flanker Task Accuracy in Pediatric Obsessive–Compulsive Disorder. *Child. Psychiatry Hum. Dev.* **1–14**10.1007/s10578-024-01711-4 (2024). 10.1007/s10578-024-01711-4 38795241

[69] Gillan, C. M., Otto, A. R., Phelps, E. A. & Daw, N. D. Model-based learning protects against forming habits. *Cogn. Affect. Behav. Neurosci.* **15**, 523–536 (2015). 25801925 10.3758/s13415-015-0347-6 PMC4526597

[70] Nebe, S., Kretzschmar, A., Brandt, M. C. & Tobler, P. N. Characterizing Human Habits in the Lab. *Collabra: Psychol.* **10**, 92949 (2024). 38463460 10.1525/collabra.92949 PMC7615722

[71] Figee, M. et al. Compulsivity in obsessive–compulsive disorder and addictions. *Eur. Neuropsychopharm*. **26**, 856–868 (2016). 10.1016/j.euroneuro.2015.12.003 26774279

[72] Robbins, T. W., Banca, P. & Belin, D. From compulsivity to compulsion: the neural basis of compulsive disorders. *Nat. Rev. Neurosci.* **25**, 313–333 (2024). 38594324 10.1038/s41583-024-00807-z

[73] Giovannelli, F. et al. Relationship between impulsivity traits and awareness of motor intention. *Eur. J. Neurosci.* **44**, 2455–2459 (2016). 27521184 10.1111/ejn.13359

[74] Peters, J. R., Erisman, S. M., Upton, B. T., Baer, R. A. & Roemer, L. A Preliminary Investigation of the Relationships Between Dispositional Mindfulness and Impulsivity. *Mindfulness* **2**, 228–235 (2011).

[75] Lu, J. & Huffman, K. A Meta-Analysis of Correlations between Trait Mindfulness and Impulsivity: Implications for Counseling. *Int. J. Adv. Couns.* **39**, 345–359 (2017).

[76] Dixon, M. R. et al. The effect of brief mindfulness training on momentary impulsivity. *J. Context Behav. Sci.* **11**, 15–20 (2019).

[77] Inzlicht, M. & Roberts, B. W. The fable of state self-control. *Curr. Opin. Psychol.* **58**, 101848 (2024). 39096668 10.1016/j.copsyc.2024.101848

[78] Settles, R. E. et al. Negative urgency: A personality predictor of externalizing behavior characterized by neuroticism, low conscientiousness, and disagreeableness. *J. Abnorm. Psychol.* **121**, 160–172 (2012). 21859164 10.1037/a0024948 PMC3299541

[79] Patton, J. H., Stanford, M. S. & Barratt, E. S. Factor Structure of the Barratt Impulsiveness Scale. *J. Clin. Psychol.* **51**, 768–774 (1995). 8778124 10.1002/1097-4679(199511)51:6<768::aid-jclp2270510607>3.0.co;2-1

[80] Dück, K., Overmeyer, R., Eger, L. & Endrass, T. Self-Reported Impulsivity Predicts Missed Study Appointments: Validating a German Adaptation of the BIS-11. *Pers. Sci.* **6**, 27000710251408750 (2025).

[81] Foa, E. B. et al. The Obsessive-Compulsive Inventory: Development and Validation of a Short Version. *Psychol. Assess.* **14**, 485–496 (2002). 12501574

[82] Gönner, S., Leonhart, R., Ecker, W. & Das Zwangsinventar obsessive-compulsive inventory-revised: a brief self-report measure for the multidimensional assessment of obsessive-compulsive symptoms]. *Psychother. Psychosom. Med. Psychol.* **57**, 395–404 (2007). OCI-R - die deutsche Version des Obsessive-Compulsive Inventory-Revised - Ein kurzes Selbstbeurteilungsinstrument zur mehrdimensionalen Messung von Zwangssymptomen [The German version of the. 10.1055/s-2007-970894 17590836

[83] *MATLAB*. The MathWorks Inc, (2021).

[84] Delorme, A. & Makeig, S. EEGLAB: an open source toolbox for analysis of single-trial EEG dynamics including independent component analysis. *J. Neurosci. Methods*. **134**, 9–21 (2004). 15102499 10.1016/j.jneumeth.2003.10.009

[85] Team, R. C. *R: A language and environment for statistical computing*. (R Foundation for Statistical Computing, at (2022). https://www.R-project.org/

[86] Pion-Tonachini, L., Kreutz-Delgado, K. & Makeig, S. ICLabel: An automated electroencephalographic independent component classifier, dataset, and website. *Neuroimage* **198**, 181–197 (2019). 31103785 10.1016/j.neuroimage.2019.05.026 PMC6592775

[87] Fischer, A. G. & Ullsperger, M. Real and Fictive Outcomes Are Processed Differently but Converge on a Common Adaptive Mechanism. *Neuron* **79**, 1243–1255 (2013). 24050408 10.1016/j.neuron.2013.07.006

[88] Benjamini, Y. & Hochberg, Y. Controlling the False Discovery Rate: A Practical and Powerful Approach to Multiple Testing. *J. Royal Stat. Soc. Ser. B Methodol.* **57**, 289–300 (1995).

[89] Cavanagh, J. F. Cortical delta activity reflects reward prediction error and related behavioral adjustments, but at different times. *NeuroImage* **110**, 205–216 (2015). 25676913 10.1016/j.neuroimage.2015.02.007

[90] Bates, D., Mächler, M., Bolker, B. & Walker, S. Fitting Linear Mixed-Effects Models Using lme4. *J. Stat. Softw.* **67**, 1–48 (2015).

[91] Hartig, F. *DHARMa: Residual Diagnostics for Hierarchical (Multi-Level / Mixed) Regression Models*. at (2024). http://florianhartig.github.io/DHARMa/

[92] Lüdecke, D. *sjPlot: Data Visualization for Statistics in Social Science*R package version 2.8.16,. at (2024). https://CRAN.R-project.org/package=sjPlot

