# Supplementary material for: Dissecting the interplay of model-based control, impulsivity and compulsivity on self-control in daily life
Source: Sci Rep. 2026 Jun 19;16:19167. doi: 10.1038/s41598-026-58046-4 (PMC13282391; doi:10.1038/s41598-026-58046-4)
Supplement: Supplementary file 1 — Supplementary Material 1 [file 41598_2026_58046_MOESM1_ESM.docx]

Supplementary material for Dissecting the interplay of model-based control, impulsivity and compulsivity on self-control in daily life

Kerstin Dück^a^, Rebecca Overmeyer^a^, Raoul Wüllhorst^a^, Tanja Endrass^a^

^a^ Faculty of Psychology, Chair for Clinical Psychology and Addiction Research, Technische Universität Dresden, 01062 Dresden, Germany

Supplement 1: Ecological momentary assessment of daily-life self-control

**Figure S1.1.** Schematic illustration of the ecological momentary assessment questionnaire on daily-life self-control.


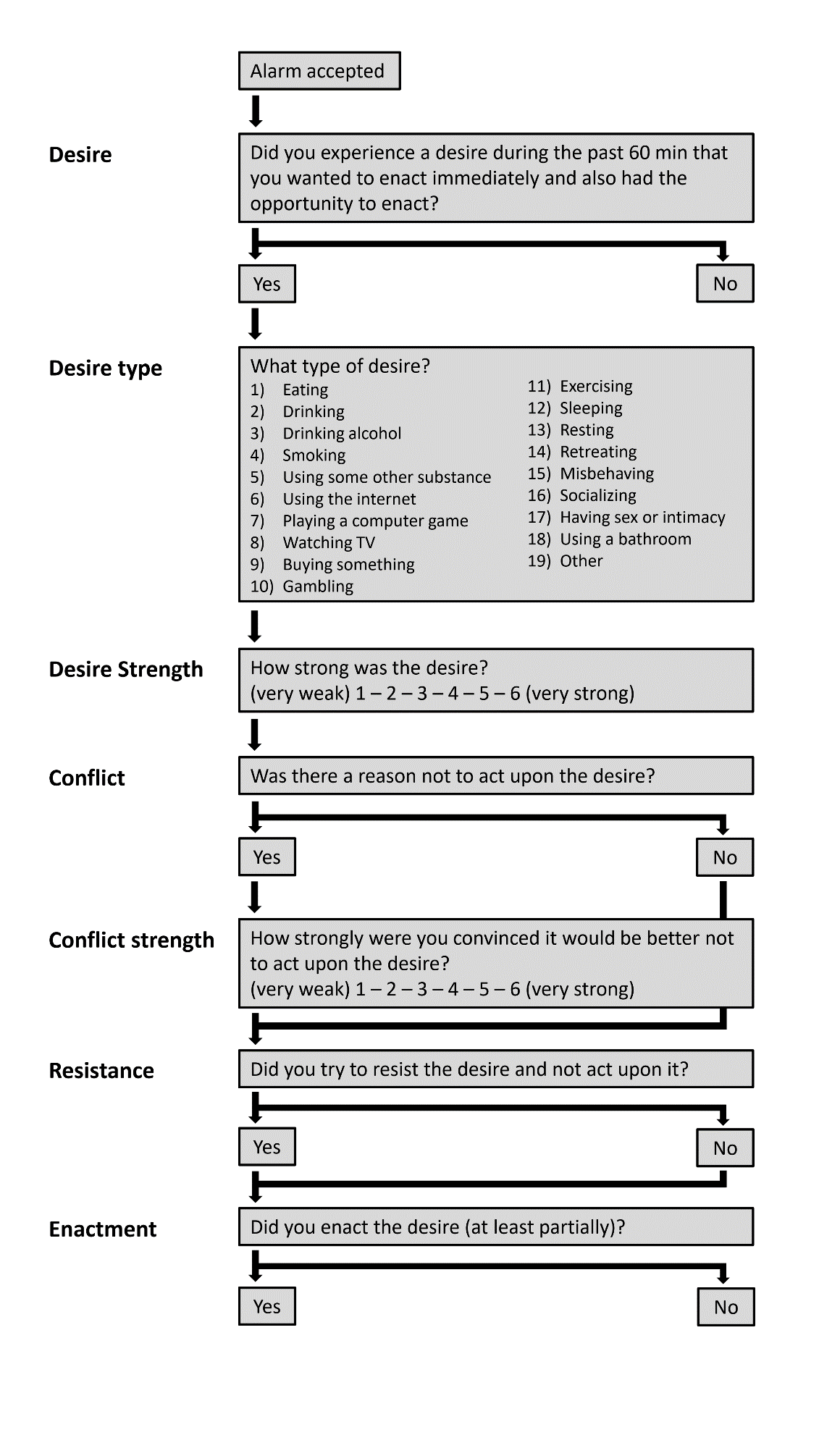


Supplement 2: Model estimation of the two-step task

Following Kool et al. [1], we compared three computational models to approximate choice behavior in our task: model-free, model based, and hybrid agents. The task involves two stages with three possible states s (stage 1: s_A_; stage 2: s_B_ or s_C_), and two possible actions a (a_A_ and a_B_). All models learn to maximize the value Q (s, a). At a given trial *t*, states are denoted as s_1,_*_t_* (always s_A_) and s_2,_*_t_* (s_B_ or s_C_), actions as a_1,_*_t_* and a_2,_*_t_*, and rewards as r_1,_*_t_* (always equal to zero) and r_2,_*_t_*.

Model-free. Model-free agents solve the task according to the SARSA(λ) temporal difference learning algorithm [2], such that at each stage i and trial t

$Q_{MF}\left( s,a \right)= Q_{MF}\left( s,a \right)+ \alpha\delta_{i,t}e_{i,t}(s,a)$.

Here, α denotes the free learning rate parameter (indicating how fast values are updated), δ*_i,t_* denotes the reward prediction error, and e*_i,t_*(s, a) denotes the free eligibility trace parameter.

As r_1,_*_t_* is always equal to zero, the first-stage reward prediction error depends on the second stage action:

$\delta_{1,t}= Q_{MF}\left( s_{2,t},a_{2,t} \right)- Q_{MF}(s_{1,t},a_{1,t})$.

The second-stage reward prediction error depends on r_2,_*_t_*:

$\delta_{2,t}= r_{2,t}-Q_{MF}(s_{2,t},a_{2,t})$.

The eligibility trace equals 0 at the beginning of each trial and is updated before the Q value according to

$e_{i,t}\left( s_{i,t},a_{i,t} \right)=e_{i-1,t}\left( s_{i,t},a_{i,t} \right)+1$.

First- and second-stage value updates occurred at the second stage. Here, prediction errors of first-stage values were weighted by the eligibility trace decay (also referred to as λ, which, if equal to zero, indicates that only values of the current stage receive an update).

Model-based. Model-based agents extend the model-free algorithm at the first stage by taking into account the transition structure P linking the first and second stages:

$Q_{MB}\left( s_{A},a_{j} \right)=P\left( s_{B} | s_{A},a_{j} \right)\max_{a\in\left\{ a_{A},a_{B} \right\}} Q_{MF}\left( s_{B},a \right)+P\left( s_{C} | s_{A},a_{j} \right)\max_{a\in\left\{ a_{A},a_{B} \right\}} Q_{MF}\left( s_{C},a \right)$.

At the second stage, model-free and model-based agents perform equivalent updates, such that Q_MF_ = Q_MB_.

Hybrid. Hybrid agents arbitrate between the Q values according to a weighting parameter w:

$Q_{net}\left( s_{A},a_{j} \right)=wQ_{MB}\left( s_{A},a_{j} \right)+(1-w)Q_{MF}(s_{A},a_{j})$.

Decision rule. Finally, Q values were subjected to a softmax function to determine choice probabilities:

$P\left( a_{i,t}=a | s_{i,t} \right)=\frac{e^{\beta(Q_{net}\left( s_{i,t},a \right)+\pi*rep\left( a \right)+\rho*resp(a))}}{\sum e^{\beta(Q_{net}\left( s_{i,t},a^{'} \right)+\pi*rep\left( a^{'} \right)+\rho*resp(a'))}}$.

Here, β indicates the stochasticity of behavior, π a choice stickiness parameter (multiplied by rep(a) = 1 if first-stage action a (stimulus choice) in the current trial was also chosen in the previous trial, otherwise zero), and a response stickiness parameter ρ (multiplied by rep(a) = 1 if the first-stage action a involved the same response key (irrespective of associated stimuli) on the current trial as in the previous trial, otherwise zero).

We estimated the free parameters (α, λ, β, *w*, π and ρ) for each model and participant individually.

We compared models excluding (pure models) and including and π and ρ .

Model fitting

We performed parameter estimation and model fit with the *fmincon* algorithm implemented in the *mfit* toolbox for MATLAB. This minimized the negative log likelihood across trials T, such that the probability of the model’s choices given a set of parameters, P(c_t_ | θ) and those of the participant converged:

$-LL= \sum_{t=1}^{T} \log P\left( c_{t} \right|\theta)$

We used Bayesian Information Criterion (BIC) for model comparison, choosing the model with the lowest mean BIC across participants, i.e. the most parsimonious fit. Starting values for the learning rate α, eligibility trace decay λ, and weighting w were drawn from flat distributions with 0 and 1 as lower and upper bounds. Inverse temperature β received starting values from gamma distribution with shape parameter = 4.82 and scale parameter = 0.88 as well as 0 and 20 as lower and upper bounds. Choice and response stickiness parameters π and ρ were drawn from normal distributions with *M*±*SD* = 0.15±1.42 and -20 and 20 as lower and upper bounds.

Fitting results

We compared the fit (mean BIC) of models with different configurations of MF, MB and hybrid learners with additional model parameters (see table S2.1). As expected, the hybrid models showed the best fit to the behavioral data. The pure hybrid model (mean BIC = 983.05) was outperformed by a model including choice stickiness (mean BIC = 929.35). However, adding response stickiness (mean BIC = 929.38) did not improve the model. The hybrid + choice stickiness model also outperformed the MF and MB models with and without choice and response stickiness and was declared the winning model.

We obtained an inverse temperature of *M*±*SD* = 4.88±1.37, a learning rate of *M*±*SD* = 0.82±0.18, an eligibility trace decay of *M*±*SD* = 0.58±0.39, and a choice stickiness of *M*±*SD* = 0.19±0.09 from this winning model.

**Table S2.1. Model fitting results**

| **Model** | | **mean BIC** |
| --- | --- | --- |
| hybrid | Pure | 983.05 |
|  | + choice stickiness | **929.35** |
|  | + choice + response stickiness | 929.38 |
| MB | Pure | 998.36 |
|  | + choice stickiness | 931.45 |
|  | + choice + response stickiness | 931.50 |
| MF | Pure | 1057.70 |
|  | + choice stickiness | 978.11 |
|  | + choice + response stickiness | 977.85 |

*Notes.* Hybrid = hybrid agent arbitrating between model-based and model-free control according to the weighting parameter *w*. MB = model-based agent. MF = model-free agent. Pure = model without any additional parameters. + choice stickiness = model including choice stickiness parameter (π). + choice + response stickiness = model including choice and response stickiness parameters (π and ρ). Mean BIC = Mean of Bayesian Information Criterion across participants.

Supplement 3: Association between inhibitory control and self-control

Inhibitory control might facilitate self-control, e.g., by inhibiting the desired response [3]. As of such, increased inhibition-related brain activity in Go/Nogo tasks has been associated with behavior requiring self-control [4]. Further research [5] has found activity in the inferior frontal gyrus (IFG), an area also related to response inhibition [6], during a Go/Nogo task to interact with contextual factors to influence self-control.

We investigated the N2 and P3a as two electroencephalographic (EEG) signals of response inhibition. The N2 is a fronto-central negative deflection peaking 200 ms after stimulus onset [7,8]. As the N2 is generally associated with conflict monitoring [9], it might influence the probability of desire enactment by altering how participants perceive conflicts between a current desire with another goal. The P3a describes a positive peak occurring fronto-centrally around 300 ms after stimulus presentation. Generally assumed to mirror attentional orienting to salient or potentially significant events [10] or as signal of (motor) inhibition [11], the P3a can be connected to the impulse inhibition aspect of self-control.

Moreover, we were interested in how possible effects of inhibition relate to impulsivity and compulsivity. Although we did not find any associations between impulsivity and event-related potentials (ERP) in a Go/Nogo task in a previous study [12], impulsivity has often been linked to altered response inhibition both behaviorally [13–16] and psychophysiologically [17,18]. For compulsivity, literature again points to a negative association to behavioral response inhibition [19,20]. Brain recordings indicate both hypoactivation [21] and hyperactivation [22] with higher compulsivity, although we could replicate neither in a Go/Nogo task [12]. We aimed to delineate how inhibitory control affects self-control and how these associations might be related to impulsivity and compulsivity.

Go/Nogo task

Response inhibition was operationalized with a Go/Nogo task consisting of 256 trials split into two blocks of 128 trials (see figure S3.1). Each trial started with a white circle presented on a black background for 200-500 ms. At the center of the circle appeared either a green square as a Go stimulus (75% of all trials) or a red square as a Nogo stimulus (25% of all trials). Participants were instructed to respond as quickly as possible to the Go stimulus with the index finger of their dominant hand and withhold their response to the Nogo stimulus. Stimuli were presented for 500 ms and were separated by a variable inter-stimulus interval of 900-1200 ms (jittered randomly in 50 ms steps; average duration was 1050 ms). Go trials were interspersed with Nogo trials, which could occur in immediate succession (only twice during the whole experiment) or be separated by up to five Go trials. Participants completed the Go/Nogo as part of an EEG session in the lab.

**Figure S3.1. Go/Nogo task**

*Notes.* Go/Nogo task used measuring response inhibition. Each trial started with a white circle (left column), then a stimulus appears in its center (middle column). Participants must respond as quickly as possible to the Go stimulus (green square, 75% of trials, top row), and withhold their response for the Nogo stimulus (red square, 25% of trials, bottom row), followed by another white circle (right column).


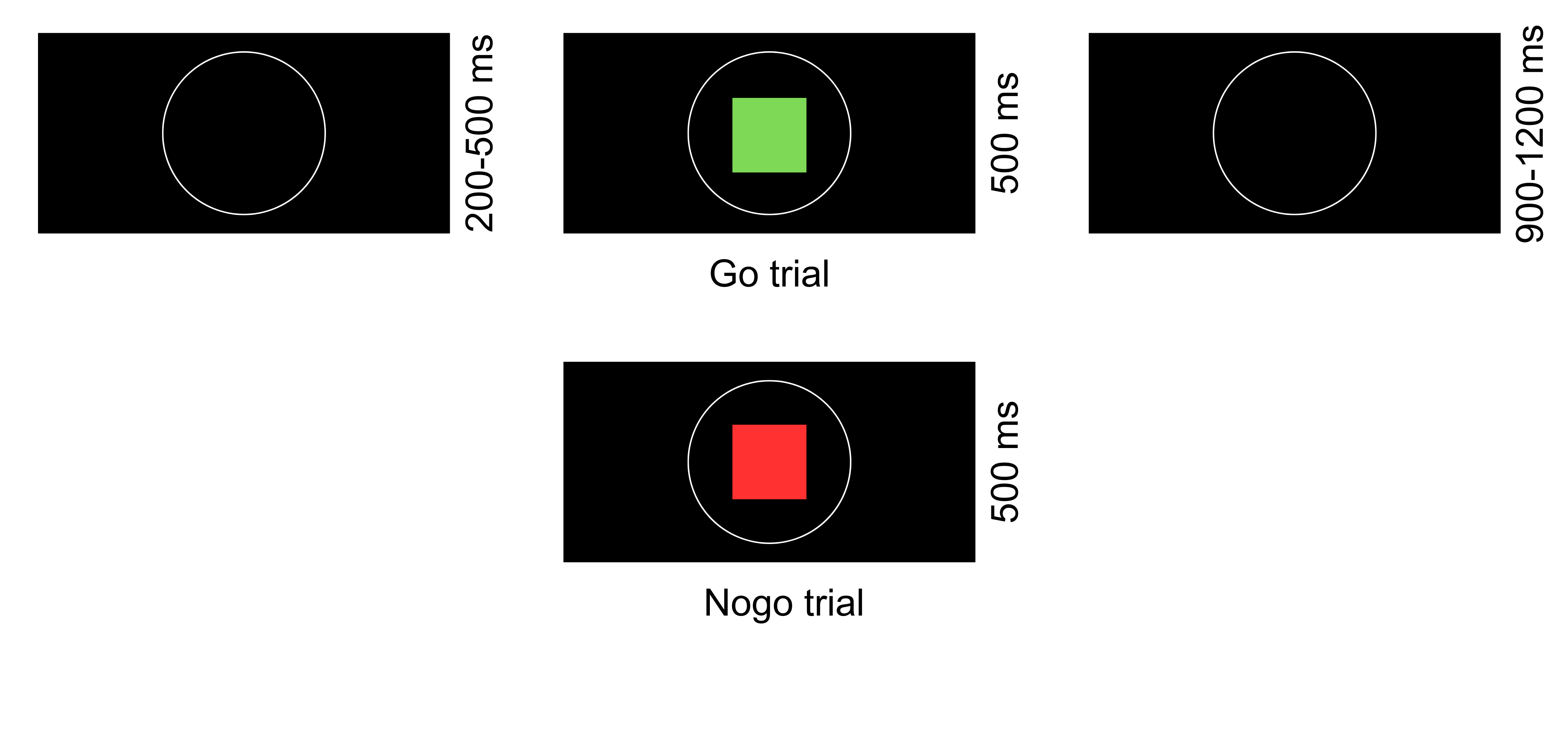


EEG recording, reduction and analysis

EEG recording and reduction for followed the protocol as described in the main manuscript. Specifically for the Go/Nogo task, baseline correction was applied in the 200 ms prior to stimulus onset. Trials including reaction times outside the range of 100 – 600 ms and failed inhibition were removed.

To establish the effects of inhibitory control, electrophysiological data was investigated in single-trial analyses to quantify the relationship between EEG activity and trial-wise characteristics. For the Go/Nogo task, stimulus-locked EEG data was used to investigate the effect of inhibition. We regressed EEG activity at each electrode and time point on trial type (Go or Nogo) using robust regression (EEG ~ Nogo stimulus). The resulting temporo-spatial maps of *b* values per subject were then averaged over subjects to investigate whether trial characteristics significantly accounted for variance in the EEG activity. We could thus compute the effect of inhibition on the EEG. As described in the main manuscript, *b* values were subjected to two-tailed one-sample t-tests against zero, employing false discovery rate (FDR) [23] to correct for multiple comparisons. We focused our analyses on the *b* values from the Go/Nogo task at electrodes and time-windows corresponding to the N2 and the stimulus-locked P3a. Based on visual inspection of the grand-averaged EEG data a from a previous publication [12], the regression effects on the N2 and the P3a were derived from Cz, where the signals appeared to be the strongest. Individual *b* value peaks were computed within the respective ERP latency ranges: for the N2, 200-300ms post-stimulus and for the stimulus-locked P3a, 300-450 ms post-stimulus [24]. To obtain individual average effects of the respective ERP for further analyses, we computed the mean of the *b* values peaks +- 20 ms for both EEG components [25].

Results: EEG task effects

We analyzed the effects of trial characteristics on the EEG data in both tasks using single-trial regression to compute a regression weight time-course for all electrodes (figure S3.2). For data from the Go/Nogo task, we observed significant positive effects around 250 ms and 350 ms after stimulus onset, with more positive EEG signals for Nogo compared to Go trials. This indicates a regression effect of successful inhibition in the time-windows of the N2 (β_mean_ = 2.82, *p* = < 0.001 at Cz) and stimulus-locked P3a (β_mean_ = 7.01, *p* = < 0.001 at Cz).

**Figure S3.2. First-level effects of single-trial regression for the Go/Nogo task**


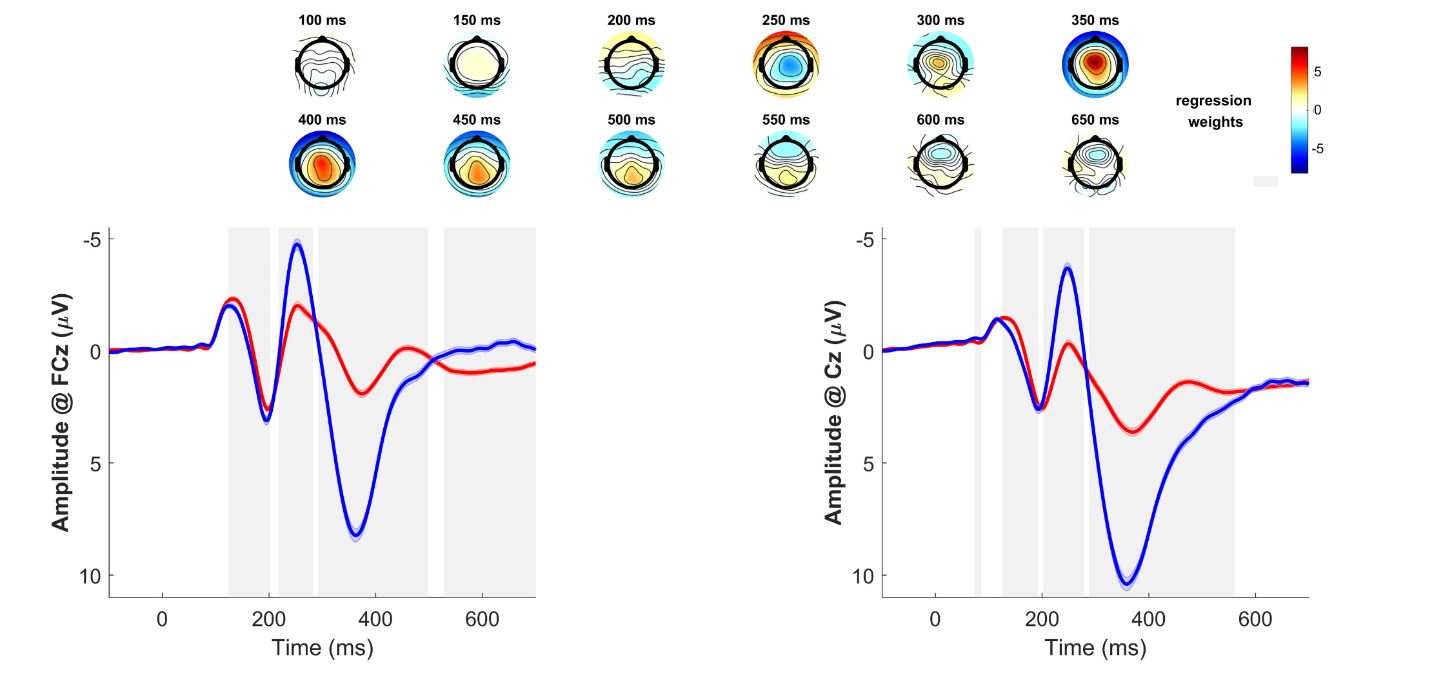


***Note.*** First-level regression of EEG data in the Go/Nogo task. First–second row: Topography of the *b* values for the first-level effect (100–650 ms) of inhibition. Third row: EEG time course at FCz (left) and Cz (right) for Go (red) vs. Nogo (blue) trials. Shading indicates SEM. EEG activity is locked to stimulus presentation. Gray shading behind EEG activity indicates significance of regression weights (*p* < .05/2) after FDR-correction.

Results: Effects on desire enactment

Based on the established interconnections, we examined the effects of inhibitory-control-related EEG activity, impulsivity and compulsivity on self-control. EEG markers of inhibitory control were computed as the individual mean *b* values of the effect of inhibition on the N2 (N2 effect) and P3a signals (P3a effect). In logistic mixed-effects models, desire enactment was then regressed onto conflict strength, desire strength, the N2 and P3a effects, as well as impulsivity and compulsivity. Desire strength and conflict strength were further modeled as random slopes to account for between-subject variability in their effects. In a second step, we included impulsivity and compulsivity scores in the model. In the full model, impulsivity and compulsivity were included as interactions with the EEG effects. In all three models, desire strength was significantly positively linked to desire enactment, while conflict strength showed a significant negative association. The N2 and P3a effects, impulsivity or compulsivity or their interactions did not significantly predict desire enactment (see tables S3.1-S3.3).

**Table S3.1. Base model predicting desire enactment with inhibitory control**

|  | **enactment** | | |
| --- | --- | --- | --- |
| Predictors | Odds Ratios | β [CI] | *p* |
| (Intercept) | 0.04 | -3.18 [-3.44, -2.92] | **<.001** |
| desire strength | 175.23 | 5.17 [4.88, 5.46] | **<.001** |
| conflict strength | 0.22 | -1.52 [-1.67, -1.37] | **<.001** |
| N2 effect | 0.92 | -0.08 [-0.27, 0.11] | .408 |
| P3a effect | 1.01 | .00 [-0.30, 0.31] | .963 |
| **Random Effects** | | | |
| σ^2^ | 3.29 | | |
| τ_00_ _name_ | 0.33 | | |
| τ_11_ _participant.desire strength_ | 2.74 | | |
| τ_11_ _participant.conflict strength_ | 0.75 | | |
| ρ_01_ | -0.64 | | |
|  | 0.35 | | |
| ICC | 0.35 | | |
| N _participant_ | 236 | | |
| Observations | 13190 | | |
| Marginal R^2^ / Conditional R^2^ | 0.648 / 0.773 | | |

*Notes.* CI = 95% confidence interval. N2 effect = mean *b* values for the inhibition effect in the time-window for the N2 at Cz. P3a = mean *b* values for the inhibition effect in the time-window for the P3a at Cz. *p* values < .05 are marked in boldface.

**Table S3.2. Second model predicting desire enactment with inhibitory control including impulsivity and compulsivity**

|  | **enactment** | | |
| --- | --- | --- | --- |
| Predictors | Odds Ratios | β [CI] | *p* |
| (Intercept) | 0.04 | -3.22 [-3.98, -2.46] | **<.001** |
| desire strength | 174.41 | 5.16 [4.87, 5.45] | **<.001** |
| conflict strength | 0.22 | -1.53 [-1.68, -1.38] | **<.001** |
| N2 effect | 0.92 | -0.08 [-0.27, 0.11] | .402 |
| P3a effect | 1.01 | 0.01 [-0.29, 0.32] | .942 |
| impulsivity | 0.93 | -0.08 [-0.79, 0.64] | .836 |
| compulsivity | 1.16 | 0.15 [-0.02, 0.33] | .089 |
| **Random Effects** | | | |
| σ^2^ | 3.29 | | |
| τ_00_ _name_ | 0.30 | | |
| τ_11_ _participant.desire strength_ | 2.75 | | |
| τ_11_ _participant.conflict strength_ | 0.75 | | |
| ρ_01_ | -0.63 | | |
|  | 0.34 | | |
| ICC | 0.36 | | |
| N _participant_ | 236 | | |
| Observations | 13190 | | |
| Marginal R^2^ / Conditional R^2^ | 0.648 / 0.774 | | |

*Notes.* CI = 95% confidence interval. N2 effect = mean *b* values for the inhibition effect in the time-window for the N2 at Cz. P3a = mean *b* values for the inhibition effect in the time-window for the P3a at Cz. Impulsivity = sum score for Barratt Impulsiveness Scale. Compulsivity = sum score for Obsessive-Compulsive Inventory-Revised. *p* values < .05 are marked in boldface.

**Table S3.3. Full model predicting desire enactment including impulsivity and compulsivity in interaction with inhibitory control**

|  | **enactment** | | |
| --- | --- | --- | --- |
| Predictors | Odds Ratios | β [CI] | *p* |
| (Intercept) | 0.04 | -3.21 [-3.47, -2.94] | **<.001** |
| desire strength | 174.52 | 5.16 [4.87, 5.45] | **<.001** |
| conflict strength | 0.22 | -1.53 [-1.67, -1.38] | **<.001** |
| N2 effect | 1.22 | 0.20 [-1.79, 2.19] | .844 |
| P3a effect | 1.33 | 0.29 [-1.73, 2.31] | .780 |
| N2 effect * impulsivity | 0.90 | -0.11 [-2.13, 1.91] | .917 |
| N2 effect * compulsivity | 0.72 | -0.33 [-2.30, 1.65] | .744 |
| P3a effect * impulsivity | 0.64 | -0.45 [-2.49, 1.59] | .664 |
| P3a effect * compulsivity | 0.75 | -0.29 [-2.37, 1.80] | .787 |
| N2 effect * impulsivity * compulsivity | 1.09 | 0.09 [-1.83, 2.00] | .928 |
| P3a effect * impulsivity * compulsivity | 1.76 | 0.57 [-1.53, 2.66] | .596 |
| **Random Effects** | | | |
| σ^2^ | 3.29 | | |
| τ_00_ _name_ | 0.31 | | |
| τ_11_ _participant.desire strength_ | 2.74 | | |
| τ_11_ _participant.conflict strength_ | 0.75 | | |
| ρ_01_ | -0.65 | | |
|  | 0.37 | | |
| ICC | 0.35 | | |
| N _participant_ | 236 | | |
| Observations | 13190 | | |
| Marginal R^2^ / Conditional R^2^ | 0.649 / 0.773 | | |

*Notes.* CI = 95% confidence interval. N2 effect = mean *b* values for the inhibition effect in the time-window for the N2 at Cz. P3a = mean *b* values for the inhibition effect in the time-window for the P3a at Cz. Impulsivity = sum score for Barratt Impulsiveness Scale. Compulsivity = sum score for Obsessive-Compulsive Inventory-Revised. *p* values < .05 are marked in boldface.

Discussion

The N2 and P3a effects as signals of inhibitory control did not predict desire enactment, nor did they significantly interact with impulsivity or compulsivity. In a previous machine-learning analysis of the Go/Nogo task [12], we found no associations between the N2 and P3as amplitude with impulsivity or compulsivity. Possibly, psychophysiological alterations come into effect only within clinical samples or in tasks that are more difficult.

Supplement 4: Behavioral and questionnaire results

**Table S4.1. Descriptive statistics for questionnaire and behavioral data**

|  |  | *M* (*SD*) | min - max |
| --- | --- | --- | --- |
|  | Impulsivity (BIS-11) | 60.58 (9.07) | 38 - 96 |
|  | Compulsivity (OCI-R) | 12.76 (9.46) | 0 - 46 |
| EMA data | Total missed EMA questionnaires | 7.37 (8.04) | 0 - 47 |
|  | Total reported desires | 34.90 (11.78) | 1- 56 |
|  | Total reported conflicts | 13.91 (8.53) | 0 - 43 |
|  | Mean desire strength | 2.74 (1.01) | 0.09 – 5.30 |
|  | Mean conflict strength | 0.97 (0.62) | 0.00 – 3.00 |
|  | Total self-control failures | 7.84 (5.86) | 0 - 30 |
|  | Ratio self-control failures | 0.16 (0.12) | 0.00 - 0.62 |
| Two-step task | Omega | 0.76 (0.22) | 0.00 - 1.00 |
|  | Inverse temperature | 4.89 (1.38) | 1.89 - 11.36 |
|  | Learning rate | 0.82 (0.18) | 0.01 - 1.00 |
|  | Eligibility trace | 0.58 (0.39) | 0.00 - 1.00 |
|  | Choice stickiness | 0.19 (0.09) | 0.04 - 0.59 |
|  | RT stage 1 (ms) | 529.06 (79.68) | 342.00 - 778.00 |
|  | RT stage 2 (ms) | 586.37 (86.89) | 347.00 - 934.00 |
| Go/Nogo task | RT Go trials (ms) | 280.95 (28.37) | 218.57 - 438.85 |
|  | RT Nogo trials (ms) | 253.16 (61.22) | 175.80 – 734.00 |
|  | Go accuracy (%) | 99 (1) | 93 - 100 |
|  | Nogo accuracy (%) | 86 (11) | 48 - 100 |

*Notes.* Impulsivity = sum score for Barratt Impulsiveness Scale 11. Compulsivity = sum score for Obsessive-Compulsive Inventory-Revised. EMA = ecological momentary assessment. Mean desire strength and mean conflict strength = mean of each participant’s mean desire and conflict ratings, unstandardized values, ranged 1 to 7. Self-control failures = enactment of a conflict-laden desire. Ratio self-control failures = enactment of conflict-laden desires divided by number of completed questionnaires. Accuracy = proportion of correct responses. Table shows raw values, variables were scaled for further regression analyses.

Supplement 5: Models predicting desire enactment in all situations with MB control

**Table S5.1. Base model predicting desire enactment with MB control**

|  | **enactment** | | |
| --- | --- | --- | --- |
| Predictors | Odds Ratios | β [CI] | *p* |
| (Intercept) | 0.04 | -3.19 [-3.14, -2.97] | **<.001** |
| desire strength | 174.79 | 5.16 [4.87, 5.45] | **<.001** |
| conflict strength | 0.22 | -1.52 [-1.67, -1.37] | **<.001** |
| FRN effect | 0.98 | -0.02 [-.18, .14] | .797 |
| P3 effect | 0.97 | -0.03 [-.17, .12] | .727 |
| **Random Effects** | | | |
| σ^2^ | 3.29 | | |
| τ_00_ _name_ | 0.32 | | |
| τ_11_ _participant.desire strength_ | 2.74 | | |
| τ_11_ _participant.conflict strength_ | 0.75 | | |
| ρ_01_ | -0.64 | | |
|  | 0.36 | | |
| ICC | 0.35 | | |
| N _participant_ | 236 | | |
| Observations | 13190 | | |
| Marginal R^2^ / Conditional R^2^ | 0.648 / 0.773 | | |

*Notes.* CI = 95% confidence interval. FRN effect = mean *b* values for transition x RPE effect in the time-window for the feedback-related negativity at FCz. P3 effect = mean *b* values for transition x RPE effect in the time-window for feedback-locked P3 at Pz. Impulsivity = sum score for Barratt Impulsiveness Scale 11. Compulsivity = sum score for Obsessive-Compulsive Inventory-Revised. *p* values < .05 are marked in boldface.

**Table S5.2. Second model predicting desire enactment with MB control including impulsivity and compulsivity**

|  | **enactment** | | |
| --- | --- | --- | --- |
| Predictors | Odds Ratios | β [CI] | *p* |
| (Intercept) | .04 | -3.19 [-3.94, -2.45] | **<.001** |
| desire strength | 173.95 | 5.16 [4.87, 5.45] | **<.001** |
| conflict strength | 0.22 | -1.53 [-1.68, -1.38] | **<.001** |
| FRN effect | 0.99 | -0.01 [-0.17, 0.14] | .878 |
| P3 effect | 0.97 | -0.03 [-0.17, 0.12] | .724 |
| impulsivity | 0.89 | -0.12 [-0.83, 0.60] | .752 |
| compulsivity | 1.16 | 0.15 [-0.03, 0.32] | .094 |
| **Random Effects** | | | |
| σ^2^ | 3.29 | | |
| τ_00_ _name_ | .29 | | |
| τ_11_ _participant.desire strength_ | 2.74 | | |
| τ_11_ _participant.conflict strength_ | 0.75 | | |
| ρ_01_ | -0.63 | | |
|  | 0.35 | | |
| ICC | 0.36 | | |
| N _participant_ | 236 | | |
| Observations | 13190 | | |
| Marginal R^2^ / Conditional R^2^ | 0.648 / 0.773 | | |

*Notes.* CI = 95% confidence interval. FRN effect = mean *b* values for transition x RPE effect in the time-window for the feedback-related negativity at FCz. P3 effect = mean *b* values for transition x RPE effect in the time-window for feedback-locked P3 at Pz. Impulsivity = sum score for Barratt Impulsiveness Scale 11. Compulsivity = sum score for Obsessive-Compulsive Inventory-Revised. *p* values < .05 are marked in boldface.

**Table S5.3. Akaike’s Information Criterion (AIC) for the models predicting desire enactment with MB control including impulsivity and compulsivity**

|  | **AIC** |
| --- | --- |
| Base model | 8140.7 |
| Second model | 8141.8 |
| Full model | 8144.9 |

*Notes.* Base model including only EEG effects. Second model adding impulsivity and compulsivity scores. Full model allowing for interactions between EEG effects and trait scores.

**Table S5.4. Model predicting desire enactment including impulsivity and compulsivity in interaction with *w* as a behavioral marker of MB control**

|  | **enactment** | | |
| --- | --- | --- | --- |
| Predictors | Odds Ratios | β [CI] | *p* |
| (Intercept) | 0.03 | -3.41 [-3.80, -3.02] | **<.001** |
| desire strength | 3.95 | 1.37 [1.30, 1.45] | **<.001** |
| conflict strength | 0.50 | -0.69 [-0.75, -0.62] | **<.001** |
| *w* | 1.72 | 0.54 [-0.71, 1.79] | .397 |
| *w* * impulsivity | 0.99 | -0.01 [-0.02, 0.01] | .566 |
| *w* * compulsivity | 0.99 | -0.01 [-0.07, 0.06] | .810 |
| *w* * impulsivity * compulsivity | 1.00 | -0.00 [-0.00, 0.00] | .539 |
| **Random Effects** | | | |
| σ^2^ | 3.29 | | |
| τ_00_ _name_ | 0.24 | | |
| τ_11_ _participant.desire strength_ | 0.21 | | |
| τ_11_ _participant.conflict strength_ | 0.17 | | |
| ρ_01_ | -0.65 | | |
|  | 0.39 | | |
| ICC | 0.35 | | |
| N _participant_ | 236 | | |
| Observations | 13190 | | |
| Marginal R^2^ / Conditional R^2^ | 0.628 / 0.759 | | |

*Notes.* CI = 95% confidence interval. Impulsivity = sum score for Barratt Impulsiveness Scale 11. Compulsivity = sum score for Obsessive-Compulsive Inventory-Revised. *p* values < .05 are marked in boldface.

Supplement 6: Models predicting conflict-laden desire enactments with MB control

**Table S6.1. Base model predicting self-control failures with MB control**

|  | **enactment** | | |
| --- | --- | --- | --- |
| Predictors | Odds Ratios | β [CI] | *p* |
| (Intercept) | 12.83 | 2.55 [1.94, 3.17] | **<.001** |
| desire strength | 8.62 | 2.15 [1.60, 2.71] | **<.001** |
| conflict strength | 0.01 | -4.51 [-5.01, -4.00] | **<.001** |
| FRN effect | 0.91 | -0.10 [-.34, .14] | .423 |
| P3 effect | 0.91 | -0.09 [-.31, .13] | .408 |
| **Random Effects** | | | |
| σ^2^ | 3.29 | | |
| τ_00_ _name_ | 2.09 | | |
| τ_11_ _participant.desire strength_ | 2.89 | | |
| τ_11_ _participant.conflict strength_ | 3.99 | | |
| ρ_01_ | -0.39 | | |
|  | -0.49 | | |
| ICC | 0.30 | | |
| N _participant_ | 231 | | |
| Observations | 3283 | | |
| Marginal R^2^ / Conditional R^2^ | 0.353 / 0.548 | | |

*Notes.* CI = 95% confidence interval. FRN effect = mean *b* values for transition x RPE effect in the time-window for the feedback-related negativity at FCz. P3 effect = mean *b* values for transition x RPE effect in the time-window for feedback-locked P3 at Pz. *p* values < .05 are marked in boldface.

**Table S6.2. Second model predicting self-control failures with MB control including impulsivity and compulsivity**

|  | **enactment** | | |
| --- | --- | --- | --- |
| Predictors | Odds Ratios | β [CI] | *p* |
| (Intercept) | 9.19 | 2.22 [1.03, 3.40] | **<.001** |
| desire strength | 8.59 | 2.15 [1.60, 2.70] | **<.001** |
| conflict strength | .01 | -4.51 [-5.01, -4.01] | **<.001** |
| FRN effect | .90 | -0.10 [-0.34, 0.14] | .419 |
| P3 effect | .91 | -0.09 [-0.31, 0.13] | .414 |
| impulsivity | 1.21 | 0.19 [-.84, 1.21] | .721 |
| compulsivity | 1.23 | 0.21 [-0.08, 0.49] | .154 |
| **Random Effects** | | | |
| σ^2^ | 3.29 | | |
| τ_00_ _name_ | 2.14 | | |
| τ_11_ _participant.desire strength_ | 2.79 | | |
| τ_11_ _participant.conflict strength_ | 3.91 | | |
| ρ_01_ | -0.36 | | |
|  | -0.51 | | |
| ICC | 0.30 | | |
| N _participant_ | 231 | | |
| Observations | 3283 | | |
| Marginal R^2^ / Conditional R^2^ | 0.356 / 0.546 | | |

*Notes.* CI = 95% confidence interval. FRN effect = mean *b* values for transition x RPE effect in the time-window for the feedback-related negativity at FCz. P3 effect = mean *b* values for transition x RPE effect in the time-window for feedback-locked P3 at Pz. Impulsivity = sum score for Barratt Impulsiveness Scale 11. Compulsivity = sum score for Obsessive-Compulsive Inventory-Revised. *p* values < .05 are marked in boldface.

**Table S6.3. Full model predicting self-control failures including impulsivity and compulsivity in interaction with MB control**

|  | **enactment** | | |
| --- | --- | --- | --- |
| Predictors | Odds Ratios | β [CI] | *p* |
| (Intercept) | 13.05 | 2.57 [1.96, 3.18] | **<.001** |
| desire strength | 8.63 | 2.16 [1.60, 2.71] | **<.001** |
| conflict strength | 0.01 | -4.49 [-5.00, -3.99] | **<.001** |
| FRN effect | 0.50 | -.69 [-2.48, 1.10] | .448 |
| P3 effect | 1.05 | .05 [-2.10, 2.20] | .962 |
| FRN effect * impulsivity | 1.72 | 0.54 [-1.14, 2.22] | .530 |
| FRN effect * compulsivity | 5.48 | 1.70 [-0.98, 4.39] | .214 |
| P3 effect * impulsivity | 0.88 | -0.13 [-2.27, 2.02] | .907 |
| P3 effect * compulsivity | 0.53 | -0.64 [-3.35, 2.08] | .646 |
| FRN effect * impulsivity * compulsivity | 0.19 | -1.65 [-4.30, 0.99] | .220 |
| P3 effect * impulsivity * compulsivity | 1.81 | 0.59 [-2.11, 3.29] | .668 |
| **Random Effects** | | | |
| σ^2^ | 3.29 | | |
| τ_00_ _participants_ | 1.84 | | |
| τ_11_ _participant.desire strength_ | 2.79 | | |
| τ_11_ _participant.conflict strength_ | 4.00 | | |
| ρ_01_ | -0.36 | | |
|  | -0.48 | | |
| ICC | 0.30 | | |
| N _participants_ | 231 | | |
| Observations | 3283 | | |
| Marginal R^2^ / Conditional R^2^ | 0.356 / 0.549 | | |

*Notes.* CI = 95% confidence interval. FRN effect = mean *b* values for transition x RPE effect in the time-window for feedback-related negativity at FCz. P3 effect = mean *b* values for transition x RPE effect in the time-window for feedback-locked P3 at Pz. Impulsivity = sum score for Barratt Impulsiveness Scale 11. Compulsivity = sum score for Obsessive-Compulsive Inventory-Revised. *p* values < .05 are marked in boldface.

Supplement 7: Predicting desire and conflict occurrence

**Table S7.1. Model predicting desire occurrence with impulsivity and compulsivity and their interactions with MB control**

|  | **Desire occurrence** | | |
| --- | --- | --- | --- |
| Predictors | Odds Ratios | β [CI] | *p* |
| (Intercept) | 3.18 | 1.16 [0.24, 2.08] | **.014** |
| Impulsivity | 0.42 | -0.87 [-1.78, 0.03] | .058 |
| Compulsivity | 1.57 | 0.45 [0.17, 0.74] | **.002** |
| FRN effect * impulsivity * compulsivity | 1.02 | 0.02 [-0.19, 0.22] | .873 |
| P3 effect * impulsivity * compulsivity | 0.89 | -0.11 [-0.32, 0.10] | .288 |
| **Random Effects** | | | |
| σ^2^ | 3.29 | | |
| τ_00_ _participants_ | 0.98 | | |
| ICC | 0.23 | | |
| N _participants_ | 236 | | |
| Observations | 13190 | | |
| Marginal R^2^ / Conditional R^2^ | 0.017 / 0.243 | | |

*Notes.* CI = 95% confidence interval. FRN effect = mean *b* values for transition x RPE effect in the time-window for the feedback-related negativity at FCz. P3 effect = mean *b* values for transition x RPE effect in the time-window for feedback-locked P3 at Pz. Impulsivity = sum score for Barratt Impulsiveness Scale 11. Compulsivity = sum score for Obsessive-Compulsive Inventory-Revised. *p* values < .05 are marked in boldface.

**Figure S7.1. Compulsivity predicts desire occurrence**


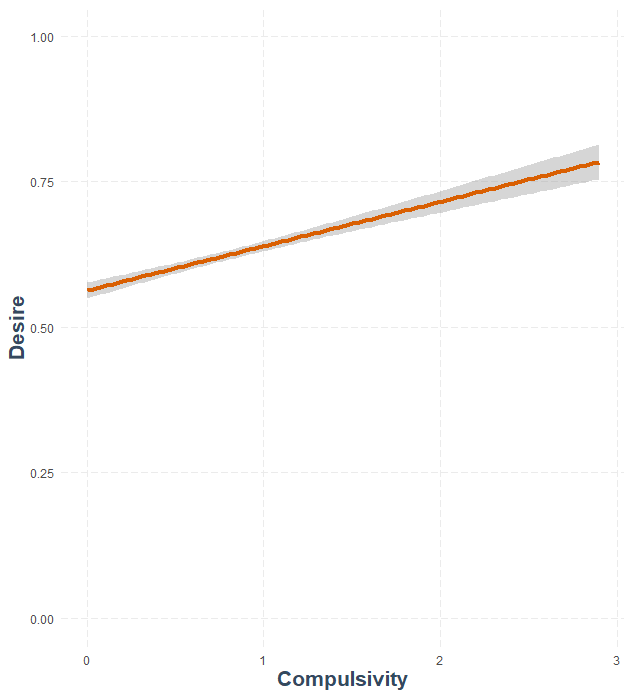


*Notes.* Regression of probability of desire occurrence compulsivity (OCI-R sum score). Shading indicates SE.

**Table S7.2. Model predicting conflict occurrence with impulsivity and compulsivity and their interactions with MB control**

|  | **Conflict occurrence** | | |
| --- | --- | --- | --- |
| Predictors | Odds Ratios | β [CI] | *p* |
| (Intercept) | 0.18 | -1.69 [-2.52, -0.87] | **<0.001** |
| Impulsivity | 1.15 | 0.14 [-0.67, 0.95] | .739 |
| Compulsivity | 1.36 | 0.31 [0.06, 0.57] | **.017** |
| FRN effect * Impulsivity * Compulsivity | 1.10 | 0.09 [-0.09, 0.28] | .332 |
| P3 effect * Impulsivity * Compulsivity | 0.94 | -0.07 [-0.25, 0.12] | .486 |
| **Random Effects** | | | |
| σ^2^ | 3.29 | | |
| τ_00_ _name_ | 0.75 | | |
| ICC | 0.19 | | |
| N _participant_ | 236 | | |
| Observations | 13190 | | |
| Marginal R^2^ / Conditional R^2^ | 0.011 / 0.195 | | |

*Notes.* CI = 95% confidence interval. FRN effect = mean *b* values for transition x RPE effect in the time-window for the feedback-related negativity at FCz. P3 effect = mean *b* values for transition x RPE effect in the time-window for feedback-locked P3 at Pz. Impulsivity = sum score for Barratt Impulsiveness Scale 11. Compulsivity = sum score for Obsessive-Compulsive Inventory-Revised. *p* values < .05 are marked in boldface.

**Figure S7.2. Compulsivity predicts conflict occurrence**


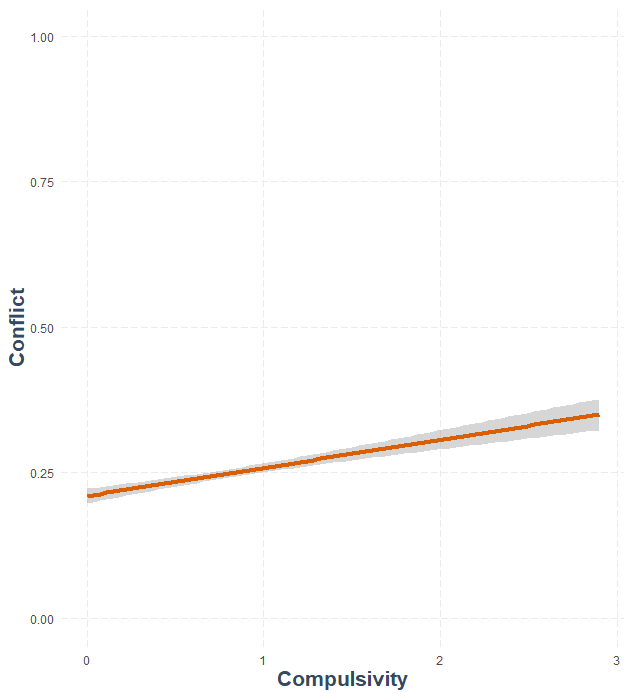


*Notes.* Regression of probability of desire occurrence compulsivity (OCI-R sum score). Shading indicates SE.

**Supplement 8. Pearson correlations between the EEG effects and impulsivity and compulsivity scores**

|  | Correlation r (*p*) | | |
| --- | --- | --- | --- |
|  | 1 | 2 | 3 |
| 1. FRN effect | - |  |  |
| 2. P3 effect | **.14** (.034) | - |  |
| 3. impulsivity | **.13** (.048) | -.01 (.923) | - |
| 4. compulsivity | -.06 (.384) | -.03 (.665) | .03 (.678) |

*Notes.* FRN effect = mean *b* values for transition x RPE effect in the time-window for the feedback-related negativity at FCz. P3 effect = mean *b* values for transition x RPE effect in the time-window for feedback-locked P3 at Pz. Impulsivity = sum score for Barratt Impulsiveness Scale 11. Compulsivity = sum score for Obsessive-Compulsive Inventory-Revised. *p* values < .05 are marked in boldface.

References

1. Kool, W., Cushman, F. A. & Gershman, S. J. When Does Model-Based Control Pay Off? *Plos Comput Biol* **12,** e1005090 (2016).

2. Rummery, G. & Niranjan, M. *On-line Q-learning using connectionist systems*. (Engineering Department, Cambridge University, 1994).

3. Baumeister, R. F. Self-regulation, ego depletion, and inhibition. *Neuropsychologia* **65,** 313–319 (2014).

4. Berkman, E. T., Falk, E. B. & Lieberman, M. D. In the Trenches of Real-World Self-Control. *Psychol. Sci.* **22,** 498–506 (2010).

5. Lopez, R. B., Hofmann, W., Wagner, D. D., Kelley, W. M. & Heatherton, T. F. Neural Predictors of Giving in to Temptation in Daily Life. *Psychological Science* **25,** 337–1344 (2014).

6. Aron, A. R., Fletcher, P. C., Bullmore, E. T., Sahakian, B. J. & Robbins, T. W. Stop-signal inhibition disrupted by damage to right inferior frontal gyrus in humans. *Nature Neuroscience* **6,** 115–116 (2003).

7. Lavric, A., Pizzagalli, D. A. & Forstmeier, S. When ‘go’ and ‘nogo’ are equally frequent: ERP components and cortical tomography. *Eur J Neurosci* **20,** 2483–2488 (2004).

8. Wessel, J. R., Danielmeier, C., Morton, J. B. & Ullsperger, M. Surprise and Error: Common Neuronal Architecture for the Processing of Errors and Novelty. *J Neurosci* **32,** 7528–7537 (2012).

9. Folstein, J. R. & Petten, C. V. Influence of cognitive control and mismatch on the N2 component of the ERP: A review. *Psychophysiology* **45,** 152–170 (2008).

10. Friedman, D., Cycowicz, Y. M. & Gaeta, H. The novelty P3: an event-related brain potential (ERP) sign of the brain’s evaluation of novelty. *Neurosci Biobehav Rev* **25,** 355–373 (2001).

11. Albert, J., López-Martín, S., Hinojosa, J. A. & Carretié, L. Spatiotemporal characterization of response inhibition. *Neuroimage* **76,** 272–281 (2013).

12. Dück, K., Overmeyer, R., Mohr, H. & Endrass, T. Are electrophysiological correlates of response inhibition linked to impulsivity and compulsivity? A machine‐learning analysis of a Go/Nogo task. *Psychophysiology* **60,** e14310 (2023).

13. Sach, M., Enge, S., Strobel, A. & Fleischhauer, M. MPQ Control (versus Impulsivity) and Need for Cognition – Relationship to behavioral inhibition and corresponding ERPs in a Go/No-Go task. *Pers Indiv Differ* **121,** 200–205 (2018).

14. Weidacker, K., Whiteford, S., Boy, F. & Johnston, S. J. Response Inhibition in the Parametric Go/No-Go Task and Its Relation to Impulsivity and Subclinical Psychopathy. *Q J Exp Psychol* **70,** 473–487 (2015).

15. Perales, J. C., Verdejo-García, A., Moya, M., Lozano, Ó. & Pérez-García, M. Bright and dark sides of impulsivity: Performance of women with high and low trait impulsivity on neuropsychological tasks. *J Clin Exp Neuropsyc* **31,** 927–944 (2009).

16. Aichert, D. S. *et al.* Associations between trait impulsivity and prepotent response inhibition. *J Clin Exp Neuropsyc* **34,** 1016–1032 (2012).

17. Shen, I.-H., Lee, D.-S. & Chen, C. The role of trait impulsivity in response inhibition: Event-related potentials in a stop-signal task. *Int. J. Psychophysiol.* **91,** 80–87 (2014).

18. Benvenuti, S. M., Sarlo, M., Buodo, G., Mento, G. & Palomba, D. Influence of impulsiveness on emotional modulation of response inhibition: An ERP study. *Clin Neurophysiol* **126,** 1915–1925 (2015).

19. Abramovitch, A., Shaham, N., Levin, L., Bar-Hen, M. & Schweiger, A. Response inhibition in a subclinical obsessive-compulsive sample. *J Behav Ther Exp Psy* **46,** 66–71 (2015).

20. Sánchez-Kuhn, A. *et al.* Go/No-Go task performance predicts differences in compulsivity but not in impulsivity personality traits. *Psychiat Res* **257,** 270–275 (2017).

21. Koorenhof, L. J. & Dommett, E. J. An Investigation Into Response Inhibition in Distinct Clinical Groups Within Obsessive-Compulsive Disorder. *J Neuropsychiatry Clin Neurosci* **31,** 228–238 (2019).

22. Kloft, L., Riesel, A. & Kathmann, N. Inhibition-related differences between tic-free and tic-related obsessive–compulsive disorder: evidence from the N2 and P3. *Exp. Brain Res.* **237,** 3449–3459 (2019).

23. Benjamini, Y. & Hochberg, Y. Controlling the False Discovery Rate: A Practical and Powerful Approach to Multiple Testing. *J Royal Statistical Soc Ser B Methodol* **57,** 289–300 (1995).

24. Enriquez-Geppert, S., Konrad, C., Pantev, C. & Huster, R. J. Conflict and inhibition differentially affect the N200/P300 complex in a combined go/nogo and stop-signal task. *Neuroimage* **51,** 877–887 (2010).

25. Kirsch, F., Kirschner, H., Fischer, A. G., Klein, T. A. & Ullsperger, M. Disentangling performance-monitoring signals encoded in feedback-related EEG dynamics. *NeuroImage* **257,** 119322 (2022).
